# Using Bayesian evidence synthesis to quantify uncertainty in population trends in smoking behaviour

**DOI:** 10.1177/09622802241310326

**Published:** 2025-02-12

**Authors:** Stephen Wade, Peter Sarich, Pavla Vaneckova, Silvia Behar-Harpaz, Preston J Ngo, Paul B Grogan, Sonya Cressman, Coral E Gartner, John M Murray, Tony Blakely, Emily Banks, Martin C Tammemagi, Karen Canfell, Marianne F Weber, Michael Caruana

**Affiliations:** 1 The Daffodil Centre, 4334 The University of Sydney, a joint venture with Cancer Council New South Wales, Kings Cross, New South Wales, Australia; 2 School of Physics, UNSW, Sydney, New South Wales, Australia; 3 Faculty of Health Sciences, 1763 Simon Fraser University, Burnaby, Canada; 4 Society for Research on Nicotine and Tobacco, 1974 The University of Queensland, Brisbane, Queensland, Australia; 5 School of Mathematics and Statistics, UNSW, Sydney, New South Wales, Australia; 6 Melbourne School of Population & Global Health, 2281 The University of Melbourne, Melbourne, Victoria, Australia; 7 National Centre for Epidemiology & Population Health, 2219 Australian National University, Canberra, Australia; 8 7497 Brock University, St Catharines, Canada; 9 Prince of Wales Clinical School, UNSW, Sydney, New South Wales, Australia

**Keywords:** Australia, Bayesian, calibration, smoking, simulation model, population trends

## Abstract

Simulation models of smoking behaviour provide vital forecasts of exposure to inform policy targets, estimates of the burden of disease, and impacts of tobacco control interventions. A key element of useful model-based forecasts is a clear picture of uncertainty due to the data used to inform the model, however, assessment of this parameter uncertainty is incomplete in almost all tobacco control models. As a remedy, we demonstrate a Bayesian approach to model calibration that quantifies parameter uncertainty. With a model calibrated to Australian data, we observed that the smoking cessation rate in Australia has increased with calendar year since the late 20th century, and in 2016 people who smoked would quit at a rate of 4.7 quit-events per 100 person-years (90% equal-tailed interval (ETI): 4.5–4.9). We found that those who quit smoking before age 30 years switched to reporting that they never smoked at a rate of approximately 2% annually (90% ETI: 1.9–2.2%). The Bayesian approach demonstrated here can be used as a blueprint to model other population behaviours that are challenging to measure directly, and to provide a clearer picture of uncertainty to decision-makers.

## 1. Background

Similar to other developed nations, the tobacco epidemic in Australia is now in its fourth stage,^1^ having peaked at an estimated 72% current smoking prevalence in the 1940s for men and 30% in the 1970s for women.^2,3^ As a signatory to the WHO Framework Convention on Tobacco Control (FCTC) and a world leader in tobacco control measures such as plain packaging, Australia has been considered a tobacco control success story, with adult current daily smoking prevalence as low as 11.6% in 2019.^4^ However, government investment in evidence-based tobacco control measures has fluctuated over time^5^ and Australia currently falls short of complying with the full suite of the WHO MPOWER policies at the highest implementation level. Since 2018, Australia has fallen out of the best-practice group in relation to taxation, mass media campaigns, and the provision of cessation services.^6^ Contemporary and reliable estimates of the impact of tobacco smoking on disease burden, health services, and societal costs are key to maintaining impetus in tobacco control. Simulation modelling studies are heavily relied upon by decision makers for these estimates; notably the U.S. Smoking History Generator from the Cancer Intervention and Surveillance Modeling Network (CISNET),^7^ SimSmoke,^8^ the Burden of Disease Epidemiology, Equity and Economics model^9^ and others (see Huang et al.^10^ and Singh et al.^11^ and references therein), however, they are prone to several sources of uncertainty that are seldom addressed.

Of 25 tobacco control policy models included in a recent review by Huang et al.,^10^ we found that only 11 reported on uncertainty. Of particular interest is ‘parameter uncertainty’, which is uncertainty in predictions due to the limitations of the data used to estimate parameter values, including sample size, sampling bias, precision, and indirect relationships between observable quantities and parameters. Structurally, almost all models assumed that uncertainties in quit and relapse rates were distributed independently. We found only one study, by Blakely et al.,^9^ that both; (a) used probability distributions to quantify parameter uncertainty for each of the key events of starting, stopping and relapsing smoking and; (b) facilitated *any* dependence between parameters. Strong or unrealistic assumptions about independence will lead to misleading estimates of uncertainty.

We propose wider use of Bayesian statistical methods as an antidote to infrequent and inconsistent reporting of parameter uncertainty among tobacco control policy models and more broadly in public health simulation modelling. The Bayesian approach to simulation model development provides a clear framework for quantifying parameter uncertainty in the form of ‘posterior distributions’, and can: (a) incorporate additional prior information along with a more detailed model of the collection of the observed data; (b) easily accommodate weaker assumptions about dependence, and; (c) is supported by a range of mature software packages, particularly in R^12^ and Python (https://www.python.org).

To aid contemporary decision-making in the Australian context, and as a demonstration of the Bayesian approach to simulation model calibration, we built a model of life-course smoking behaviours for the Australian population in the pre-vape era using nationally representative data on smoking status collected between 1962 and 2016. The pre-vape era is an important baseline with extensive data for future modelling of the impact of any disruptions. We complement and extend the existing tutorials on Bayesian calibration^13–16^ with concise, and more complete, steps from parameterisation to (posterior) prediction. With the calibrated model, we describe estimates of the historical trends in key quantities of smoking behaviour in Australia; the proportion that initiated smoking in birth cohorts born between 1910 and 1996; the rate of quitting smoking between 1930 and 2016, and; the rate that individuals who smoked early in life switched to reporting as never smoking when surveyed later in life. This latter quantity has never been estimated using smoking data from cross-sectional health surveys. Furthermore, we estimate uncertainty allowing for dependence between all parameters; throughout, we report 90% equal-tailed intervals (ETIs) to demonstrate that descriptions of uncertainty are not confined to 95% confidence intervals, which may be helpful when engaging with decision-makers.

## 2. Methods

The structure of our model was based on a previous Australian model of population-wide smoking behaviours by Gartner et al.^17^ that used data 1983–2007. We extended the observation period using individual-level data from 26 cross-sectional surveys conducted 1962–2016. In an earlier analysis of the 26 surveys, Vaneckova et al.^3^ identified trends in smoking prevalence at younger ages consistent with an effect where those who formerly smoked switched to reporting as having never smoked. We updated the original model to accommodate this transition. We also replaced estimates of ‘net cessation’ in the model with ‘permanent cessation’ using clear assumptions about relapse.

The calibration process was as follows:

(1) The parameters to be calibrated were determined by examination of *structural identifiability* (see Cole^18^).
(2) The model parameters were assigned prior distributions (‘priors’), which included hazard ratio (HR) estimates of death from a cohort study.
(3) A statistical model of survey sampling and responses was constructed.
(4) A suitable neighbourhood of parameter-values from which to sample was identified via analysis of a measure of ‘practical identifiability’ introduced by Raue et al.^19^
(5) 200 samples of parameter values were obtained using a Markov Chain Monte Carlo algorithm for each of eight models under consideration.
(6) The best model was selected based on Deviance Information Criterion (DIC) and summary statistics of estimated ‘model inadequacy’ as defined by Kennedy and O’Hagan.^20^

Using the sample of parameter values we quantified historical trends in life-course smoking behaviours by the corresponding sample of: the expected proportion that initiated smoking; the expected smoking cessation rate, and; the expected rate that those who formerly smoked switched to reporting that they never smoked.

In the following subsections we describe the model using its real-world context, we describe the data sources, and we outline the choices made and algorithms used in each step of the calibration and to compute the outputs. Throughout, references are provided to Supplemental Material for more technical detail: which includes equations that may assist some readers.

### 2.1. Model structure

**Figure 1.**
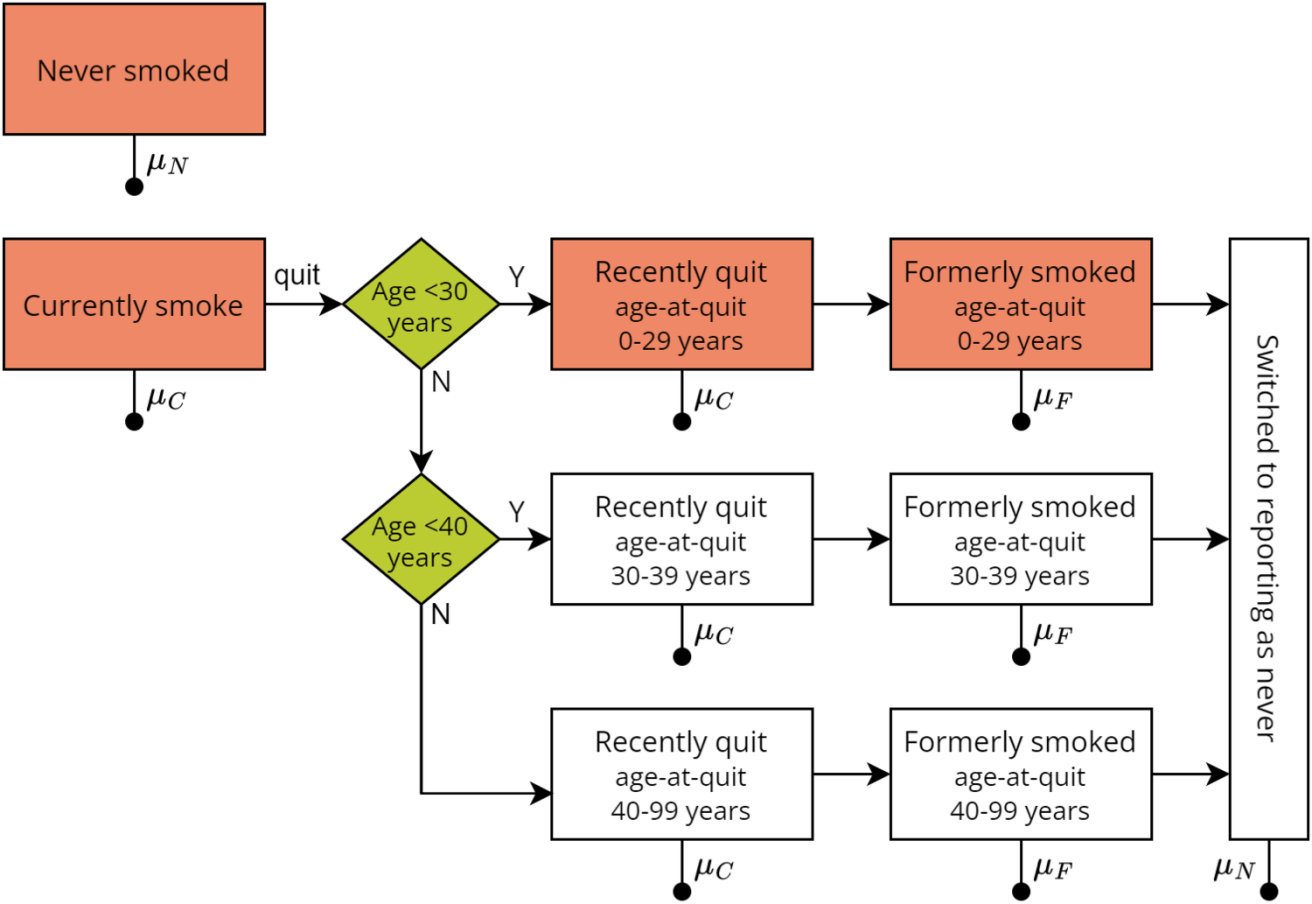
Structure of the compartmental model of lifetime smoking behaviour. Line with filled circle represents transition to death. Transition rates between boxes depend upon age and sex, except for the transition from having recently quit to formerly smoked which occurs at two years in the recently-quit state. $\mu_{N}$ is the never-smoked mortality rate. $\mu_{C}={HR}_{C}\mu_{N}$ and $\mu_{F}={HR}_{F}\mu_{N}$ , where ${HR}_{C}$ and ${HR}_{F}$ are the hazard ratios of death for those who currently smoke and those who formerly smoked, respectively, compared to those who never smoked.

We used a compartmental model of the lifetime smoking behaviour of an individual (or cohort), summarised in Figure 1. The life-course of an individual proceeded in the following order:

1. An individual either started smoking before age 20 years, or they did not smoke throughout their adult lifetime.
2. If they started smoking;
  - they would eventually quit and enter a ‘recently quit’ state, according to their age-at-quit group;
  - after 2 years they entered a ‘formerly smoked’ state according to their age-at-quit group, and;
  - they may switch to reporting as having never smoked, i.e. enter the ‘reporting-as-never’ state.

A quit event was defined as the event that an individual stopped smoking daily/regularly and did not smoke again. No explicit model of quit *attempts* and relapse was included. The conditions required for consistent estimates of permanent-quit rates given the data sources discussed below are stated in Supplemental Material Appendix A.

We assigned an age-at-quit category so that those who smoked for a short duration may transition to reporting-as-never at a different rate from those who smoked for longer.

The starting age in the simulation was 20 years, where each individual starts in either the ‘never’, ‘currently’, ‘recently-quit’, or ‘former’ smoking category. The assumptions governing the initial proportion in each category are provided in Supplemental Material Section B.1.

At all times, an individual may die at a rate specific to their smoking status, sex, and piece-wise constant by age:

- Individuals who never smoked and reported-as-never died at the same rate, denoted $\mu_{N}$ .
- Individuals who currently smoked and those who recently quit died at the rate $\mu_{C}={HR}_{C}\times \mu_{N}$ , with age-specific hazard ratio ${HR}_{C}$ .
- Individuals who formerly smoked died at the rate $\mu_{F}={HR}_{F}\times \mu_{N}$ , with age-specific hazard ratio ${HR}_{F}$ .

We assumed the population mortality rate μ (age, sex and year-specific) satisfied:

(1) $$\mu =\mu_{N}\rho_{N}+\mu_{C}\rho_{C}+\mu_{F}\rho_{F}$$

where $\rho_{N}$ was the (population) proportion in the union of the never smoked and reporting-as-never categories, $\rho_{C}$ was the proportion in the union of the currently smoke and recently quit categories, and $\rho_{F}$ was the proportion in the union of the formerly smoked categories.

For a mathematical description of the compartmental model shown in Figure 1 and described above, see Supplemental Material Section B.1. The population proportions for each category of smoker were computed from the mathematical description, as outlined in Supplemental Material Section B.1.1, provided values of the model-parameters were specified.

### 2.2. Model parameterisation

The two key quantities for the initial population at age 20 years were the proportion of individuals that formerly smoked (amongst current and former), denoted $P_{F}$ , and the proportion that initiated, denoted $P_{I}$ (one minus the proportion that never smoked). We modelled the logit-transformed proportion that initiated as a natural cubic spline-function of the birth year. Recall that the initial size of each category is formulated in Supplemental Material Section B.1.

The quit rate, which we defined as the rate amongst those who smoked of quit events as described above, was denoted as $\lambda_{Q}$ (also the rate of exit from the ‘currently smoke’ state in Figure 1), and we modelled the log-transformed value as the sum of natural cubic spline-functions of age and calendar year. We assumed that the rate of transition between the formerly smoked category to the reporting-as-never state, denoted $\lambda_{R}$ , for each age-at-quit group (<30 years, 30–39 years, ≥ 40 years) was constant.

We pre-specified the domain of each spline and the location of their knots, for the precise specification see Supplemental Material Section B.2. To test different models, we used differing numbers of knots, which were sequentially included to form eight different models, including a ‘null’ model with only intercept terms and no reporting-as-never (see Supplemental Material Section B.3 and Table B1). We assumed that the total number of degrees of freedom, including the age-group specific HRs, would be small compared to the number of observations in the surveys so that the parameters might be practically identifiable in each model.

### 2.3. Data sources

#### 2.3.1. Smoking status

Data on population-level smoking behaviour were obtained from nationally representative surveys. The Australian Data Archive and Cancer Council Victoria (CCV) provided; the National Drug Strategy Household Surveys (NDSHS) and selected predecessors 1985–2016 (Australian Institute of Health and Welfare^21^ and McAllister^22^ and ref. therein); the Risk Factor Prevalence Study (RFPS) 1980–89 (Risk Factor Prevalence Study Management Committee^23^ and ref. therein); the CCV Australian adult smoking surveys 1974–95 (Hill et al.^24^ and ref. therein), and; selected Australian Gallup Polls 1962–67.^25–28^ While additional data were available for 2019,^4^ we did not include these in the model to eliminate structural uncertainty in the vape era, thus our model can, in future, be used to compare observed prevalence in the post-vape era to a counter-factual no-vaping scenario. We have previously reported the sample size, collection method, response rate, weighting approach, and other details about the surveys in Vaneckova et al.^3^

Details about how we categorised the smoking status of participants are provided in Supplemental Material Appendix A. In brief, smoking status was defined as ‘currently smoking’ for participants who smoked on a daily/regular basis depending on the survey questionnaire (see Vaneckova et al.^3^), with the remainder of non-missing responses classified as not actively smoking. In all surveys except those of the Gallup Polls, participants were further categorised as those who ‘recently quit’, ‘formerly smoked’, or ‘never smoked’. For participants with sufficient information on a quit event, we further divided those who formerly smoked or recently quit into age-at-quit categories.

The surveys contained 254,231 observations in total. We singly-imputed age in years for those whose age-at-survey was grouped using a Penalised Composite Link Model fitted to each individual survey (i.e. 60,131 participants had their age imputed).^29^ We included participants of age 20–99 years at the time of the survey, born from 1910 onwards (229,581 participants). We excluded 3400 observations for missing smoking status, leaving a total of 226,181 observations. Participants who had quit smoking but had missing age-at-quit were assigned to ‘formerly smoked’.

Non-random sampling occurred in the surveys, either due to a multi-stage stratified or quota-sampling design, or due to non-response bias. All surveys supplied weights (except those from the AGP in 1967, the CCV surveys in 1974 and 1980, the National Campaign Against Drug Abuse and Social Issues Survey in 1991 and the additional Victorian sample of the NDSHS in 1995) which were designed to match the weighted count of responses to Australian population data by age, sex and in some cases, other demographic variables. We applied an iterative proportional fitting procedure^30,31^ to all surveys to estimate a correction to the supplied weights such that the re-weighted response counts matched population counts by age, sex, state, and capital city versus non-capital city residence.^32,33^

#### 2.3.2. Mortality

Australian population mortality data by age and sex for the period 1930–2016 was obtained from the Human Mortality Database.^34^

#### 2.3.3. Hazard ratios of mortality by smoking status

We estimated smoking-status specific HRs of death from the 45 and Up Study, a prospective cohort study of 267,153 residents of New South Wales aged ≥ 45 years at baseline (2006–2009), randomly sampled from the Services Australia (formerly the Department of Human Services and Medicare Australia) enrolment database.^35^ People ≥ 80 years of age and residents of rural and remote areas were oversampled by design, and the overall response rate was about 18% of invitees. Fact of death to August 2017 was ascertained from the NSW Registry of Births Deaths and Marriages, with data linkage performed by the NSW Ministry of Health’s Centre for Health Record Linkage (CHeReL; www.cherel.org.au). Ethics approval for the 45 and Up Study was provided by the University of New South Wales (UNSW) Human Research Ethics Committee (reference: HC210602) on 25 July 2005 (with regular extensions granted, most recently from 9 September 2021 to 8 September 2026), and for this specific analysis by the NSW Population Health Services Research Ethics Committee (reference: 2014/08/551). All participants consented to participate by signing a consent form that accompanied the questionnaire. All procedures were performed in accordance with the Declaration of Helsinki.

We applied Cox proportional hazards regressions separately by sex for each 5-year age stratum from 45 to 99 years to estimate HRs and 95% confidence intervals (CI) of death in relation to smoking status at baseline (‘currently smoking’ – including those who had quit within 2 years of baseline – and ‘formerly smoked’, relative to ‘never smoked’) using previously reported methods by Banks et al.^36^ Briefly, participants were excluded from analysis if at baseline they were aged ≥ 100 years, had data linkage errors, had a self-reported history of chronic disease (heart disease, stroke, blood clot, and cancer except melanoma and non-melanoma skin cancer), and/or missing information on smoking status. HRs were adjusted for age. After exclusions, 191 031 participants were included for analysis. Ethics approval for the 45 and Up Study was provided by the University of NSW Human Research Ethics Committee, and approval for data linkage by the CHeReL was provided by the NSW Population and Health Services Research Ethics Committee.

To assess the sensitivity of the model predictions to the selection of priors for the HRs, we conducted a sensitivity analysis whereby we calibrated the selected model using estimates of HRs derived from the 12-year follow-up of the CPS-I cohort,^37^ recruited in the United States (US) 1959–1960, as the prior instead of the 45 and Up Study estimates. Given that the estimates of the HRs have increased over time in the US,^38^ this was a prior that greatly under-estimated smoking-related mortality in the later period of the smoking survey data.

### 2.4. Bayesian calibration

#### 2.4.1. Parameters to calibrate

An initial step in model calibration, Bayesian or otherwise,^39^ is to determine for which parameters would values be estimated, thus we informally examined *structural identifiability*.^18^ In brief, structurally identifiable parameters are those that *can* be calibrated with observations of the modelled process, while structurally non-identifiable parameters will require other data sources or observations. We proposed that the quantities $P_{F}$ and $P_{I}$ could be identified from the observed proportions at the starting age. The remaining four quantities, $\lambda_{Q}$ , $\lambda_{R}$ , $\mu_{C}$ , and $\mu_{F}$ , were not identifiable from observed proportions, but if any one of these were known, the remaining three would be identifiable. Details are provided in Supplemental Material Section B.4. Therefore, if these four parameters were all to be quantified via calibration, at least one would need to have an informative prior, rather than a non-informative prior.

#### 2.4.2. Prior distribution

We assumed that the prior of the parameters could be decomposed into independent priors for; the HRs, the proportion $P_{F}$ ; the coefficients of the spline describing $P_{I}$ (including an intercept); the coefficients of the sum of the splines in $\lambda_{Q}$ (including an intercept), and; each rate of switching to reporting-as-never.

We used an informative prior for the HRs, given by the asymptotic distribution of the estimate from the Cox regressions (see Section 2.3). In doing so, we addressed the non-identifiability described above by supplying information on both $\mu_{C}$ and $\mu_{F}$ via equation (1) and the integration routine outlined in Supplemental Material Section B.1.1. Although our examination of structural identifiability suggested we only needed prior information on one of these two, there was no reason to leave additional readily available information out of the calibration process.

The proportion $P_{F}$ was given a uniform prior on [0,0.5] , indicating indifference to any value in that range. We assigned data-dependent multivariate normal priors to the coefficients of $P_{I}$ and $\lambda_{Q}$ , centred at no effect and intended to have minimal impact on their posterior distribution compared to the impact of the observed data. One nuisance parameter was introduced for each data-dependent prior. We assigned each rate of switching to reporting-as-never, $\lambda_{R}$ , the prior $1/\sqrt{\lambda_{R}}$ . See Supplemental Material Section B.5 for further details about the values of the parameters of the prior distribution.

#### 2.4.3. Model of smoking survey responses

We constructed a statistical model of the ‘observation process’ and a mapping between the parameters of the model and the parameters of the observation process. The dependence structure of the priors, the model, the smoking survey data, and the population mortality data are shown in Supplemental Material Figure B2. To obtain a maximum a priori (MAP) estimate of the parameter values and to sample parameter values within this structure (i.e. calibrate the model) we derived an expression for likelihood of the smoking survey data.

We assumed each survey response was drawn from a categorical distribution, with sex-, age-, and birth year-specific probabilities. The three categories were named ‘current’, ‘former’ (by age-at-quit group), and ‘never’, with definitions as per above. We assumed the probabilities for each category were the same as the sum of one or more expected proportions in the model. The ‘current’ probabilities were given by the sum of the model’s ‘currently smoking’ and ‘recently-quit’ proportions; ‘former’ probabilities were given by the model’s ‘former smoking’ proportions; and ‘never’ probabilities were the remainder. We found the expected proportions for the model by numerically solving its governing equations (see Supplemental Material Section B.1).

Uncertainty introduced by non-random sampling was accounted for using the effective sample size of each cell in the cross-tabulation of each survey by sex, age, and birth year. The likelihood for each independent cell was given by a Dirichlet distribution with parameters determined by observed weighted proportions and the effective sample size (see Supplemental Material Section B.7).

#### 2.4.4. Suitable sampling region

There is less risk of non-convergence when practical identifiability holds in the parameter space. We found a suitable region to sample from by investigating local practical identifiability of the MAP estimate of the parameters. For this step, only, we fixed the HRs at their prior mode. We defined local practical identifiability as the posterior-based 95% CI of the estimate being finite in extent.^19^ We used the *optim()* command in R to find the MAP estimate.^12^ We calculated the profile posterior of each component using a predictor-corrector approach (see Supplemental Material Section C.1), in the neighbourhood given by ± 7.1 standard deviations of the asymptotic distribution of the MAP estimate. We then estimated the highest level of confidence for which the CI was contained within the neighbourhood; if the level was less than 95% then practical identifiability of the parameter near the MAP estimate was questionable.

The degree to which the HRs were identified by the survey data was investigated with the overlap statistic^40,41^ which was calculated using the approach outlined in Supplemental Material Section C.2 and the posterior sample obtained in the next step; the threshold 0.35 or less was used as evidence of weak identifiability, as suggested by Garrett and Zeger.^40^

#### 2.4.5. Parameter-value samples

We used the Metropolis-within-Gibbs algorithm to sample from the joint posterior of the HRs, the model parameters, and the nuisance parameters. Each of these was the basis of a block in the Gibbs sampler. Five chains were simulated, starting at random points drawn from a distribution over-dispersed with respect to the asymptotic distribution of the estimate of the HRs and the MAP estimate of the other parameters. After a burn-in phase of length 1600 samples per chain, the samples were discarded and the algorithm resumed until an estimated effective sample size of 50 was obtained (see Gelman et al.^42^ equation 11.8). We culled each chain to 40 evenly-spaced samples, for a total of 200 samples of parameter values (see Supplemental Material Appendix D). We used this procedure for each of the eight models listed in Supplemental Material Table B1.

#### 2.4.6. Model selection

We estimated the DIC for each model to assess improvement in predictive accuracy between models. The procedure to calculate the DIC using the samples from the joint posterior is detailed in Supplemental Material Section E.1. We also considered summary measures of the model discrepancy, or inadequacy, (defined by Kennedy and O’Hagan^20^ as the difference between the model’s expected value and corresponding observed data) in the proportion of ‘currently smoking’; the proportion of ‘never smoked’ among those not ‘currently smoking’; and the proportion of ‘formerly smoked’ who had quit before age 30 years. The discrepancy was summarised using the expected mean and standard deviation on a log-odds scale of the discrepancy for the observed survey data; with the expectation taken over the parameter-posterior. We applied some discretion in selecting the final model if improvement in the DIC or discrepancy over a preceding model was small to reduce risk of poorer out-of-sample predictive performance (‘over-fitting’).

### 2.5. Cross-sectional smoking prevalence

We generated 200 samples of the predicted proportions of individuals with never, current, and former smoking status for each survey by sex and calendar year using Dirichlet-Multinomial random variables (see Supplemental Material Section B.7). Each sample corresponded to one prediction from the model using one of the samples of the parameter values. We compared the observed proportions in the NDSHS 2016 to model predictions via the probabilities that each model prediction was more extreme than the observed value, i.e. the ‘two-tailed’ statistic 2min(q,1−q) where q is the quantile at the observed value. The probabilities were estimated using the corresponding proportions in the sample.

### 2.6. Trends in life-course of smoking

We generated 200 samples of;

- the expected proportion who initiated smoking before age 20 years by birth year and sex;
- the expected rate of quitting smoking by age, calendar year, and sex, and;
- the expected rate that individuals who formerly smoked would switch to reporting-as-never in a survey by sex and age-at-quit group.

Predictions for the cross-sectional prevalence were obtained using the (set of) expected proportions generated above, the effective sample size, and the distribution assumed for the survey in (see Supplemental Material Section B.7). In total, 90% ETIs of these outputs were given by the 5th and 95th percentiles over the 200 samples obtained for each.

The quit rate could not be validated directly using the survey data as the quit event in our model was conditional upon no future relapse, and we did not know which individuals in a survey would relapse. However, evidence suggests that 95% of those with at least two years abstinence, maintain abstinence over the next year,^43^ therefore we compared the model to an estimate of the quit rate from two years prior to a survey given sustained abstinence of at least two years.

### 2.7. Sensitivity analyses

We tested the sensitivity of the selected model’s predictions to different choices in calibration. We tested: (a) the prior for the HRs of death in relation to smoking status; (b) the age at which initiation of smoking was completed within a cohort; (c) using a cohort term in the quit rate as opposed to a calendar year term; (d) whether those who quit after age 40 years could report as never smoking; (e) allowing any individual who formerly smoked to report as never; and (f) whether survey weights were used in the likelihood (details in Supplemental Material Sections F.1 to F.5).

## 3. Results

### 3.1. Calibration and model selection

The neighbourhood of the MAP estimate for each model met our requirements for a suitable region for sampling parameter-values. The Hessian of the posterior function at the MAP estimate was positive definite for each model, therefore the neighbourhoods all contained a local maximum. Practical identifiability in the neighbourhood was supported by the confidence-level test in all but a few cases. Notable exceptions were; the nuisance parameters, for which the highest confidence level varied between 65% and 98% depending on the model, and; the parameter describing the rate that men who formerly smoked switched to reporting-as-never smoked, with age-at-quit 30–39 years, for which the level was no greater than 70% in any model. The highest confidence level for which the corresponding interval was contained within the computed neighbourhood is provided for each model in Table E2 (Supplemental Material). Mortality HRs did not meet the criteria of weak identifiability (as expected) according to the overlap statistic; the distributions of the prior mortality HRs and the posterior HRs are summarised by their median and 5th and 95th percentiles in Table E3 (Supplemental Material), along with the overlap statistics.

The parameter-value samples from the MCMC algorithm satisfied our criteria regarding (non-) convergence. All samples reached an effective sample size of at least 50.0, and the estimated potential variance reduction was at most 1.11 for any component after 1710 iterations per chain.

Based on the DIC estimates for each tested model, as shown in Table E4 (Supplemental Material), the model selected for the main analysis had three degrees of freedom in the effect of birth year in the proportion of a cohort that initiated smoking; two degrees of freedom each for the effects of age and calendar-year in the smoking quit rate; and non-zero rates of switching to reporting as never smoked by age-at-quit group for those who quit before age 40 years (model ‘F’ in Supplemental Material Table E4).

The measures of discrepancy of the selected model were much smaller than those for the ‘null’ model. The magnitude of the mean discrepancy – a measure of bias – in either the proportions of individuals in the population that smoke or the proportions of individuals that never smoked amongst those not currently smoking, both on a log-odds scale, was at most 0.024 in the selected model, compared to at most 0.170 for the ‘null’ model. Likewise, the standard deviation – a measure of incomplete variation – was no greater than 0.153 in the selected model, compared to at most 0.497 in the ‘null’ model. The magnitudes of the mean and the standard deviations of the discrepancy in the proportion of individuals that formerly smoked who had quit prior to age 30 years were greater than for the other proportions (for each model), with values for the selected model at most 0.114 and 0.409 respectively. The mean and standard deviation in the discrepancy for each model and each proportion evaluated is shown in Supplemental Material Table E5.

### 3.2. Model estimates of trends in smoking behaviour

#### 3.2.1. Cross-sectional smoking prevalence

**Figure 2.**
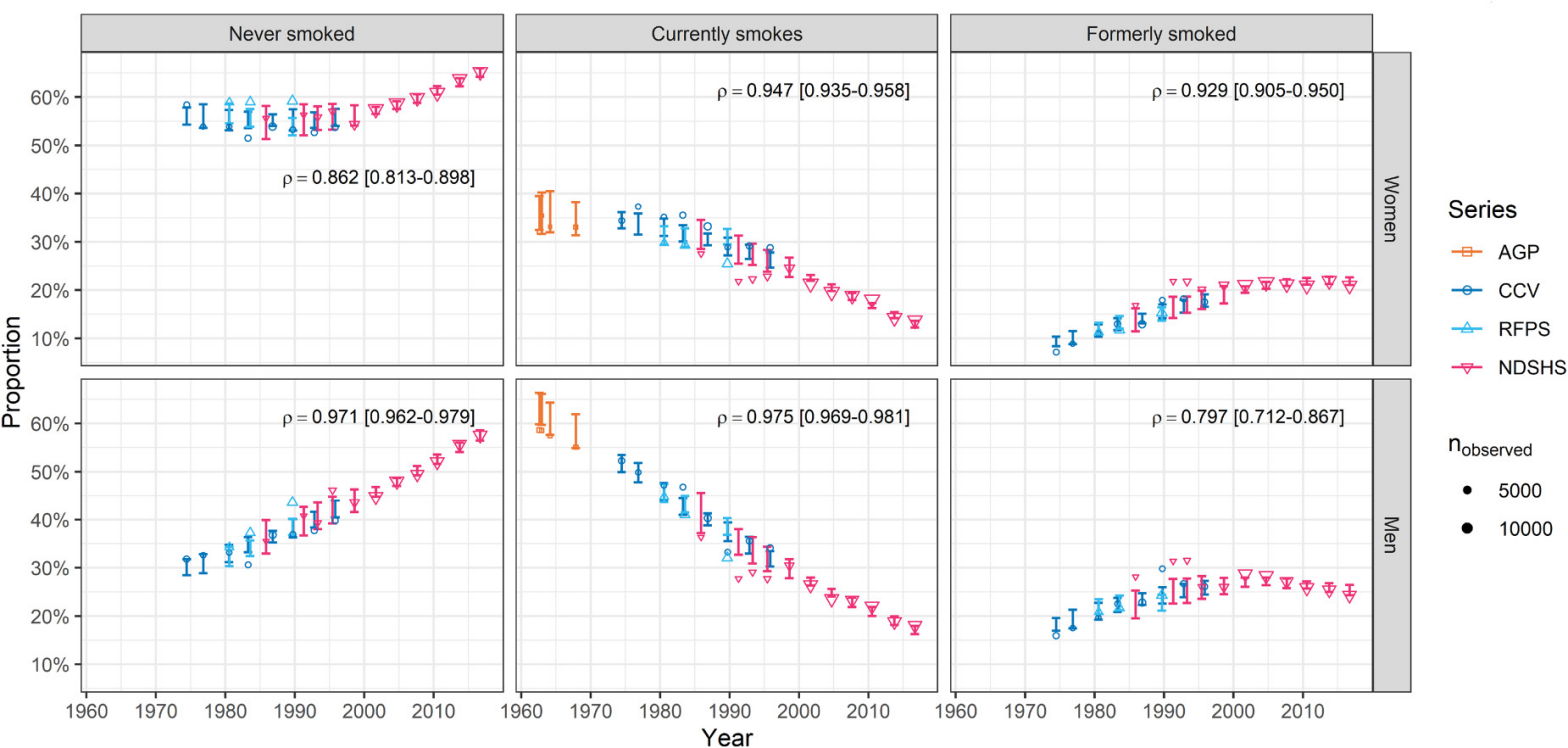
The observed proportion of Australian men and women aged ≥ 20 years by smoking status (current/former/never) in four smoking survey series conducted between 1962 and 2016 (markers), and corresponding osterior predictive intervals from the model (error bars) given by the 5th and 95th percentiles of the sample we obtained via Markov Chain Monte Carlo. Size of marker indicates number of respondents aged ≥ 20 years in the survey in the category, $n_{\text{observed}}$ . Median Pearson correlation between survey data and the model-samples (and interval given by 5th and 95th percentiles) shown on each panel. AGP: Australian Gallup Polls; CCV: Cancer Council Victoria adult smoking survey; RFPS: Risk Factor Prevalence Study; NDSHS: National Drug Strategy Household Survey.

The predicted cross-sectional proportion of Australian men and women aged ≥ 20 years in each smoking status category is shown by survey in Figure 2. Overall, 52.7% of the survey proportions were within the 90% ETIs (indicating some under-coverage). The Pearson correlations between the survey data and the model-sampled predictions are also presented. These were lower for the quantities with more non-linear calendar year trends and higher for simpler trends (e.g. 90% of correlations for the proportion of men that currently smoked were in the interval 0.969–0.981).

The proportions of participants in the NDSHS in 2016 by smoking status and the corresponding 90% ETIs from the model are shown in Table 1, along with the estimate of the p -value that predictions were more extreme than the survey value. The p -values for the proportion that never smoked were all equal or greater than 0.800. The p -values for the proportions of those who currently and formerly smoked were no less than 0.080 for men, and no greater than 0.070 for women or persons.

#### 3.2.2. Initiation, cessation, and reporting smoking status

The sample obtained from the model of the proportion of Australians that initiated daily smoking by 10-year birth cohorts from 1910 to 1996 is summarised in Table 2. Uptake peaked for women born in 1962, with just over half of all women born at that time estimated to have initiated smoking (sample median 55.4%, 90% ETI 54.8%–56.1%). Since the peak, the proportion decreased and for women born in 1996, the sample median was 16.2% (90% ETI: 15.3%–17.2%). For men, uptake was highest in the earliest cohort (1910), with 87.4% (90% ETI: 86.1%–88.6%) estimated to have initiated smoking, and decreased with each successive birth cohort to 22.4% (90% ETI: 21.0%–24.0%) for those born in 1996.

**Table 1.** Distribution of respondents ≥ 20 years of age in the 2016 National Drug Strategy Household Survey by smoking status, and corresponding posterior predictions sampled from the model via Markov Chain Monte Carlo, for men, women and persons.

Sex ( n )	Smoking status	Observed proportion	Model sample	p -value
*Median [5th,95th percentile]*			
**Women (12 120)**	Never	65.2%	65.1% [64.2%,66.0%]	0.80
	Current	13.8%	12.9% [12.2%,13.6%]	0.03
	Former	21.0%	21.9% [21.1%,22.7%]	0.07
**Men (10 032)**	Never	57.6%	57.5% [56.4%,58.6%]	0.93
	Current	18.1%	17.2% [16.3%,18.0%]	0.08
	Former	24.3%	25.3% [24.3%,26.5%]	0.12
**Persons (22 152)**	Never	61.5%	61.4% [60.7%,62.1%]	0.86
	Current	15.9%	15.0% [14.4%,15.5%]	≤ 0.01
	Former	22.6%	23.6% [23.0%,24.3%]	≤ 0.01

The p -value is the proportion of the model-sampled predictions that were more extreme than the observed value in the survey.

**Table 2.** Model estimate of the proportion and annual growth rate in the proportion of Australians that initiated smoking among men, women, and persons born in selected single years between 1910 and 1996.

Birth year	Women	Men	Persons	p -value: M ≡ W
*Proportion initiated smoking (Median [5th,95th percentile])*		
**1910**	43.9% [42.4%,45.6%]	87.4% [86.1%,88.6%]	66.4% [65.4%,67.5%]	≤ 0.001
**1920**	43.1% [42.1%,44.2%]	82.5% [81.4%,83.5%]	63.2% [62.5%,63.9%]	≤ 0.001
**1930**	43.5% [42.8%,44.4%]	76.7% [75.8%,77.6%]	60.4% [59.8%,61.0%]	≤ 0.001
**1940**	46.4% [45.7%,47.3%]	70.9% [70.0%,71.6%]	59.0% [58.5%,59.5%]	≤ 0.001
**1950**	51.8% [51.1%,52.4%]	65.7% [65.0%,66.3%]	58.9% [58.4%,59.3%]	≤ 0.001
**1960**	55.3% [54.7%,56.0%]	60.0% [59.4%,60.6%]	57.7% [57.3%,58.2%]	≤ 0.001
**1970**	52.2% [51.6%,52.9%]	52.0% [51.3%,52.6%]	52.1% [51.7%,52.6%]	0.830
**1980**	40.1% [39.5%,40.7%]	40.8% [40.2%,41.6%]	40.5% [40.1%,41.0%]	0.154
**1990**	24.2% [23.3%,25.1%]	28.8% [27.7%,30.1%]	26.6% [25.8%,27.4%]	≤ 0.001
**1996**	16.2% [15.3%,17.2%]	22.4% [21.0%,24.0%]	19.4% [18.4%,20.3%]	≤ 0.001
*Annual growth in proportion initiated smoking (Median [5th,95th percentile])*		
**1910**	− 0.23% [ − 0.41%, − 0.03%]	− 0.49% [ − 0.52%, − 0.46%]	− 0.37% [ − 0.47%, − 0.27%]	0.028
**1920**	− 0.09% [ − 0.27%,0.09%]	− 0.66% [ − 0.72%, − 0.60%]	− 0.38% [ − 0.48%, − 0.29%]	≤ 0.001
**1930**	0.34% [0.21%,0.45%]	− 0.78% [ − 0.85%, − 0.71%]	− 0.23% [ − 0.31%, − 0.16%]	≤ 0.001
**1940**	0.99% [0.91%,1.05%]	− 0.77% [ − 0.81%, − 0.72%]	0.09% [0.04%,0.13%]	≤ 0.001
**1950**	1.01% [0.92%,1.08%]	− 0.79% [ − 0.85%, − 0.72%]	0.10% [0.04%,0.15%]	≤ 0.001
**1960**	0.17% [0.11%,0.23%]	− 1.09% [ − 1.16%, − 1.01%]	− 0.47% [ − 0.52%, − 0.42%]	≤ 0.001
**1970**	− 1.54% [ − 1.61%, − 1.46%]	− 1.88% [ − 1.95%, − 1.79%]	− 1.71% [ − 1.76%, − 1.66%]	≤ 0.001
**1980**	− 3.84% [ − 4.03%, − 3.65%]	− 2.95% [ − 3.17%, − 2.72%]	− 3.39% [ − 3.55%, − 3.23%]	≤ 0.001
**1990**	− 6.17% [ − 6.51%, − 5.78%]	− 3.96% [ − 4.35%, − 3.56%]	− 5.03% [ − 5.31%, − 4.75%]	≤ 0.001
**1996**	− 7.09% [ − 7.48%, − 6.64%]	− 4.40% [ − 4.87%, − 3.94%]	− 5.71% [ − 6.03%, − 5.38%]	≤ 0.001

Shown are the sample median and the interval given by the 5th and 95th percentiles from the posterior sample obtained via Markov Chain Monte Carlo. The p -value is the mean proportion of more extreme values for men compared to women.

The sample obtained from the model of the quit rate per 100 person-years (PY) amongst Australians who smoke daily is summarised in Table 3. The sampled quit rates increased with calendar year throughout. The effect of age varied by sex, where in a given calendar year, women quit at higher rates at earlier and later ages compared to age 50 years. For example, in the last year of the calibration period, 2016, the median sampled quit rate per 100PY for women at age 30 and 70 years was 5.52 (90% ETI: 5.18–5.86) and 5.89 (90% ETI: 5.22–6.48), respectively, whereas at age 50 years, the sample median was 5.08 (90% ETI: [4.80,5.30]) events per 100PY. Whereas for men, the quit rate increased with age in a fixed calendar year. That is, the median sampled quit rate per 100PY for men at age 30 years in 2016 was 3.66 (90% ETI: 3.44–3.90), whereas at age 70 years was 4.78 (90% ETI: 4.16–5.35). The quit rate for women grew faster than for men through the years 1930 until 2016 (Table 4). In 2016, the quit rate over all age groups for men who smoke was 4.1 events per 100PY (90% ETI: 3.9–4.4), the rate for women was 5.4 events per 100PY (90% ETI: 5.1–5.7), and for men and women (combined) who smoke it was 4.7 events per 100PY (90% ETI: 4.5–4.9).

**Table 3.** Model estimate of the rate that Australians quit daily smoking (per 100 person-years) obtained from the selected model by sex, selected ages (30, 50 and 70 years), and ten-year increments of calendar year from 1940 to 2010 and in 2016.

	Age 30 years			Age 50 years			Age 70 years		
Calendar year	Women	Men	p -value	Women	Men	p -value	Women	Men	p -value
**1940**	0.33 [0.27,0.43]	0.50 [0.43,0.57]	0.015	–	–	–	–	–	–
**1950**	0.61 [0.53,0.72]	0.82 [0.74,0.90]	0.008	–	–	–	–	–	–
**1960**	1.07 [0.99,1.17]	1.29 [1.22,1.36]	0.004	0.98 [0.91,1.06]	1.66 [1.58,1.73]	≤ 0.001	–	–	–
**1970**	1.73 [1.65,1.81]	1.87 [1.79,1.93]	0.037	1.59 [1.51,1.65]	2.40 [2.32,2.47]	≤ 0.001	–	–	–
**1980**	2.53 [2.44,2.61]	2.45 [2.37,2.52]	0.269	2.32 [2.22,2.40]	3.14 [3.05,3.25]	≤ 0.001	2.70 [2.39,2.92]	3.19 [2.82,3.53]	0.060
**1990**	3.37 [3.27,3.45]	2.94 [2.85,3.02]	≤ 0.001	3.09 [2.96,3.18]	3.77 [3.66,3.87]	≤ 0.001	3.58 [3.19,3.86]	3.83 [3.39,4.22]	0.463
**2000**	4.19 [4.08,4.29]	3.30 [3.18,3.40]	≤ 0.001	3.84 [3.72,3.95]	4.23 [4.11,4.35]	≤ 0.001	4.46 [3.96,4.82]	4.29 [3.80,4.74]	0.655
**2010**	5.00 [4.78,5.22]	3.54 [3.37,3.72]	≤ 0.001	4.60 [4.41,4.76]	4.55 [4.36,4.77]	0.795	5.34 [4.72,5.81]	4.62 [4.05,5.15]	0.141
**2016**	5.52 [5.18,5.86]	3.66 [3.44,3.90]	≤ 0.001	5.08 [4.80,5.30]	4.71 [4.46,5.02]	0.157	5.89 [5.22,6.48]	4.78 [4.16,5.35]	0.052

Shown are the sample median and the interval given by the 5th and 95th percentiles of the posterior sample obtained via Markov Chain Monte Carlo. p -value; the mean proportion of more extreme values for men compared to each value for women.

**Table 4.** Model estimate of the growth in the smoking quit rate of Australians by sex, and ten-year increments of calendar year from 1930 to 2010 and in 2016.

Calendar year	Women	Men	p -value
**1930**	6.40% [5.47%,7.23%]	5.28% [4.71%,5.96%]	0.092
**1940**	6.26% [5.37%,7.06%]	5.14% [4.60%,5.79%]	0.079
**1950**	5.86% [5.08%,6.57%]	4.75% [4.28%,5.32%]	0.048
**1960**	5.22% [4.61%,5.77%]	4.12% [3.74%,4.54%]	0.012
**1970**	4.31% [3.93%,4.65%]	3.24% [3.01%,3.48%]	≤ 0.001
**1980**	3.28% [3.13%,3.44%]	2.22% [2.09%,2.34%]	≤ 0.001
**1990**	2.49% [2.32%,2.63%]	1.43% [1.25%,1.57%]	≤ 0.001
**2000**	1.95% [1.70%,2.19%]	0.90% [0.66%,1.13%]	≤ 0.001
**2010**	1.66% [1.36%,1.97%]	0.62% [0.34%,0.90%]	≤ 0.001
**2016**	1.61% [1.30%,1.93%]	0.57% [0.29%,0.86%]	≤ 0.001

Shown are the sample median and the interval given by the 5th and 95th percentiles of the posterior sample obtained via Markov Chain Monte Carlo. p -value; the mean proportion of more extreme values for men compared to each value for women.

People switched smoking status from former to reporting-as-never at higher rates if they quit earlier. The median sampled rate for those who quit before age 30 years was 2.05 (90% ETI: 1.92–2.18) switch-to-reporting-as-never events per 100PY, and for those who quit between ages 30 and 39 years it was 0.29 (90% ETI: 0.14–0.45) events per 100PY.

The sample of the proportion of those who started smoking before age 20 years who had also quit smoking by age 20 was 8.6% (90% ETI: 7.2%–9.8%) for men and 8.0% (90% ETI: 6.8%–9.2%) for women. The estimate of the quit rate from three years prior to a survey (given sustained abstinence of at least two years) compared to the model’s quit rate is shown in Supplemental Material Figure E3. The survey estimate was, on average, marginally higher than the model, which may be partly explained by relapse after two years amongst some individuals.

### 3.3. Sensitivity analyses

Detailed results of each sensitivity analysis are in Supplemental Material Tables G6 to G11. The models in each sensitivity analysis were less compatible with the data (greater value of DIC) compared to the selected model, except for the marginal improvement in DIC when women who had quit after the age of 40 years were allowed to switch to reporting-as-never. The proportion that initiated smoking did not appear sensitive to the source of prior smoking-related mortality HRs, the use of a nonlinear cohort effect in the quit rate (as opposed to calendar year), nor allowing those who quit after age 40 years to switch to reporting-as-never. There were small increases in the proportion initiated in the sensitivity analyses where: no switch to reporting-as-never was allowed; the survey weights were ignored, and; the age at completed initiation was raised to 25 years.

The sample of the quit rate was most sensitive to the use of a non-linear cohort effect in the model of the quit rate compared to a calendar year effect (main analysis), resulting in a greater quit rate across all ages and for both men and women; however this was less compatible with the survey data than the main analysis according to the DIC. The sample of the quit rate at younger and older ages was also sensitive to raising the age at completed initiation from 20 to 25 years, which resulted in greater quit rates in the sample. For all the other variations in the models tested, the effects were mostly seen at older ages, with one exception. The estimated quit rate at younger ages decreased marginally if the switch to reporting-as-never was not allowed. At older ages, the quit rate increased for both men and women when the prior mortality HR by smoking status was taken from the CPS-I study. Amongst women, but not men, at older ages, the estimated quit rate increased marginally if assumptions about the switch to reporting-as-never were varied. The sample of the rate of switching to reporting-as-never was sensitive to raising the age at completed initiation, increasing marginally for those who had quit prior to age 30 years. The posterior HR was consistent with the main analysis except for a marginal increase in the HR for those that formerly smoked when no switch to reporting-as-never was allowed.

## 4. Discussion

Our Australian smoking behaviour model is the first cohort-specific model of life-course smoking behaviours calibrated using Bayesian statistics. The discrepancy between the observed and modelled smoking prevalence was < 2.3% over the whole study period. In recent years, fewer Australians within a birth cohort started daily smoking than at any time over the past 80 years. This is also the first study to estimate long-term historical trends in smoking cessation rates by age, sex, birth cohort and calendar year in Australia. The rate that people quit daily smoking increased with every successive birth cohort, however by 2016, women were quitting at a higher rate than men across all age groups.

We utilised 26 surveys 1962–2016 to identify age-, sex-, and calendar year-specific trends in permanent quit-event rates. In settings where there are fewer surveys available, the effects may need to be simplified in order to satisfy identifiability requirements. For example, the calendar year effect may be simplified to a step function at a particular *a priori* known year where policy was imposed. The practical identifiability analysis we performed could be a useful blueprint for determining when simplifications to the model are needed due to sparse or scarce data.

Our model allowed those that had quit smoking to report as having never smoked, which is one of the mechanisms that may explain increases in the never-smoked proportion observed at early ages within a cohort.^3^ This transition was more frequent for those who quit smoking before age 30 years than those who quit later in life. We speculate that it would also be associated with those who had smoked occasionally or at a lower intensity. The inclusion of this pathway decreased the discrepancy in the proportion of those who quit at younger ages, and was more compatible with the data than a model without this pathway. Removing this pathway decreased the estimated proportion that initiated smoking and increased the quit rate at middle or older ages. There is some evidence that this event’s rate is near zero amongst men aged 30–39 years. Other models may obtain better estimates of the initiation rates and quit rates by including this effect.

We modelled the discrepancy between observed and predicted outcomes in a similar, but not identical, fashion to that of Kennedy and O’Hagan,^20^ and we estimated it with a widely available and efficient procedure.^44^ We described bias and unexplained variance by age and cohort for a more diverse set of outcomes than was used in both the Australian model by Gartner et al.^17^ upon which this work was based, and the US CISNET Smoking History Generator (i.e. expected proportions of; current-smoking vs non-smoking, former-smoking as a proportion of non-smoking, and proportion who quit smoking before age 30 years).^7^ Our approach may be a useful blueprint for assessing model performance in a manner that mutes the effects of aleatory uncertainty; in our example this means the uncertainty due to the random selection of a finite number of survey and study participants.

The estimated discrepancy in the proportion of those who formerly smoked and had quit before age 30 years suggested the model was biased towards earlier ages at quitting. This could be caused by an over-estimated quit rate, or an insufficient model of the switch to reporting-as-never. We are not aware of any assessment of this in other smoking behaviour models, and therefore it is unclear if this bias is considered a significant problem. If it is indeed a problem, then one could, rather than revising the model and calibrating again, incorporate the estimate of the discrepancy into predictions.^45^

We found that 53% of the observed cross-sectional proportion of participants by smoking status (never/current/former smoking) were contained in the 90% ETIs. This finding could be caused by either an imprecise model of the survey data, for example our ambivalence to between-survey effects, selection bias that was not alleviated via our use of iterative proportional fitting, or that the model is lacking some detail or effect. There is visual evidence of between-survey effects in Figure 2, and these may be driven by one or more of the following; changes in delivery and sequences of questions, different participation and response rates, or changes in the questionnaire which could cause inconsistent categorisation of smoking status (see Supplemental Material Appendix A). The under-coverage of the prediction intervals for the surveys could be addressed with more explicit (or hierarchical) modelling of these factors; we propose this would only marginally improve accuracy of our statements made about the population.

We present here a comprehensive outline for a generalisable Bayesian approach to calibrate a model of population-wide behaviours. We did this by bringing together key elements from several previous introductions and tutorials for Bayesian calibration.^13–16^ The calibration methodology incorporated identifiability analyses, Bayesian evidence synthesis of multiple data sources, and uncertainty quantification. This provided the following advantages over earlier smoking behaviour model calibration efforts: (a) we could show that model predictions were measurably improved by allowing those who had quit smoking long-term to switch to reporting as having never smoked; (b) we quantified uncertainty in trends of smoking initiation and cessation due to the random sampling in the surveys and trials used to calibrate the model; and (c) we accounted for competing events exactly as all transitions were calibrated together.

The modelling approach had some structural limitations. (a) We did not consider interactions between calendar year and age in cessation rates, meaning we could not examine differences in the long-term effects of social norms and tobacco policy between age groups. (b) Those who quit at younger ages have a lower risk of smoking-related mortality than those who quit at later ages, however our model did not vary the hazard ratio of mortality by age-at-quit, therefore our estimate of the quit rate may be underestimated at older ages and overestimated at younger ages. (c) We did not account for the effect of migration in and out of the population. Differences in smoking prevalence between immigrants and emigrants, combined with enough migration, would bias the estimates of the rates and the model predictions.^17^ (d) Earlier cohorts of women initiated smoking later^3^ and this could cause underestimation of the quit rate at younger ages in earlier cohorts; although this bias appeared mild in sensitivity analyses.

## 5. Conclusions

We have provided a step-by-step approach to Bayesian calibration of a simulation model of smoking behaviour in Australia, which can form a blueprint for modelling in other jurisdictions where decision makers need reliable accounting of uncertainty. Our calibration synthesised multiple data sources including 26 representative smoking surveys, population mortality data, and a large Australian cohort study, the 45 and Up Study. Using the model we demonstrated that the proportion of a cohort that initiate smoking has declined from earlier peaks, that smoking cessation rates have been increasing, and that those who quit smoking at earlier ages may have switched to reporting that they have never smoked in surveys. Furthermore, unlike many models, we provided estimates of the level of uncertainty that policy-makers can consider. This model can be used to forecast smoking prevalence in the Australian population and assess the potential impact of new or ongoing interventions in tobacco control.

## Supplemental Material

sj-pdf-1-smm-10.1177_09622802241310326 - Supplemental material for Using Bayesian evidence synthesis to quantify uncertainty in population trends in smoking behaviour Supplemental material, sj-pdf-1-smm-10.1177_09622802241310326 for Using Bayesian evidence synthesis to quantify uncertainty in population trends in smoking behaviour by StephenWade, Peter Sarich, Pavla Vaneckova, Silvia Behar-Harpaz, Preston J Ngo, Paul B Grogan1, Sonya Cressman, Coral E Gartner, John M Murray, Tony Blakely, Emily Banks, Martin C Tammemagi, Karen Canfell1, Marianne FWeber and Michael Caruana in Medical Research
