# Supplementary material for: Using Bayesian evidence synthesis to quantify uncertainty in population trends in smoking behaviour
Source: Stat Methods Med Res. 2025 Feb 12;34(3):545–60. doi: 10.1177/09622802241310326 (PMC11951451; doi:10.1177/09622802241310326)
Supplement: sj-pdf-1-smm-10.1177_09622802241310326 - Supplemental material for Using Bayesian evidence synthesis to quantify uncertainty in population trends in smoking behaviour [file sj-pdf-1-smm-10.1177_09622802241310326.pdf]

Using Bayesian evidence synthesis to quantify uncertainty in population trends in smoking behaviour

Statistical Methods in Medical Research
XX(X):2–29
©The Author(s) 2024
Reprints and permission:
sagepub.co.uk/journalsPermissions.nav
DOI: 10.1177/ToBeAssigned
www.sagepub.com/
SAGE

Stephen Wade[1], Peter Sarich[1], Pavla Vaneckova[1], Silvia Behar-Harpaz[2], Preston J Ngo[1], Paul B Grogan[1], Sonya Cressman[3], Coral E Gartner[4], John M Murray[5], Tony Blakely[6], Emily Banks[7], Martin C Tammemagi[8], Karen Canfell[1,9], Marianne F Weber[1,†], Michael Caruana[1,†]

Abstract

Simulation models of smoking behaviour provide vital forecasts of exposure to inform policy targets, estimates of the burden of disease, and impacts of tobacco control interventions. A key element of useful model-based forecasts is a clear picture of uncertainty due to the data used to inform the model, however, assessment of this parameter uncertainty is incomplete in almost all tobacco control models. As a remedy, we demonstrate a Bayesian approach to model calibration that quantifies parameter uncertainty. With a model calibrated to Australian data, we observed that the smoking cessation rate in Australia has increased with calendar year since the late 20th century, and in 2016 people who smoked would quit at a rate of 4.7 quit-events per 100 person-years (90% equal-tailed interval [ETI]: 4.5-4.9). We found that those who quit smoking before age 30 years switched to reporting that they never smoked at a rate of approximately 2% annually (90% ETI: 1.9%-2.2%). The Bayesian approach demonstrated here can be used as a blueprint to model other population behaviours that are challenging to measure directly, and to provide a clearer picture of uncertainty to decision-makers.

Keywords
Australia, Bayesian, calibration, smoking, simulation model, population trends

Supplementary Material

Appendix A Smoking surveys

The surveys provided by the Australian Data Archive were:

- National Drug Strategy Household Survey (NDSHS) 1998, 2001, 2004, 2007, 2010, 2013, and 2016 (see [1] references therein).
- National Campaign Against Drug Abuse and Social Issues Survey (NCADASIS) 1991 and 1993 (including the Victorian Drug Household Survey 1993 sample), NDSHS 1995 (including the Victorian Drug Strategy Household Survey 1995 sample), Social Issues Australia (SIA) survey 1985 (see [2] references therein).
- Risk Factor Prevalence Study (RFPS) 1980, 1983, and 1989 (see [3] references therein).
- Cancer Council Victoria (CCV) Australian adult smoking surveys in 1974, 1976, 1980, and 1983 (see [4] references therein).
- Australian Gallup Polls (AGP) no. 158, 160, 168 and 193 (1962-1967)[5–8].

The CCV Australian adult smoking surveys in 1976, 1986, 1989, 1992, and 1995 were obtained under agreement with Cancer Council Victoria.

A flow chart that summarised the categorisation of respondents across all surveys into separate smoking behaviour categories is shown in Figure A1. The most comprehensive questions were contained in the 2001 NDSHS and onwards, for this survey the key questions used to categorise smoking status were:

[1]The Daffodil Centre, The University of Sydney, a joint venture with Cancer Council New South Wales, [2]School of Physics, UNSW, [3]Faculty of Health Sciences, Simon Fraser University, [4]Society for Research on Nicotine and Tobacco, The University of Queensland, [5]School of Mathematics and Statistics, UNSW, [6]Melbourne School of Population & Global Health, The University of Melbourne,[7]National Centre for Epidemiology & Population Health, Australian National University, [8]Brock University, [9]Prince of Wales Clinical School, UNSW.
† Joint senior author.

Corresponding author:
Stephen Wade, Daffodil Centre, PO Box 572, Kings Cross, NSW 1340.
Email: stephen.wade@sydney.edu.au

1. "Have you ever smoked on a daily basis? (mark one response)"
    - "Yes, I smoke now."
    - "Yes, I used to smoke daily, but not now."
    - "No, never smoked daily."
2. "About what age were you when you stopped smoking daily?" (YY)

These questions were mapped to the flow chart and respondents were assigned categories accordingly. Note that those who did not respond to the first question were assigned missing smoking status. Those who had quit smoking but whose age-at-quit was missing were assigned to 'formerly smoked', given that the clear majority (86%) of complete cases had quit at least two years prior. The rate of missing information on whether participants who did not currently smoke had smoked in the past or had quit within two years was less than 8.3% in all but the 1985-95 NDSHS and the 1989 CCV survey. The 1985-95 NDSHS did not include the relevant questions, while only a subset of participants in the 1989 CCV survey who formerly smoked were asked how long ago they had stopped.

The same questions, with a marginally different sequence were asked in 1998, however in the earlier surveys from 1985-1995 the key questions on smoking behaviour were:

1. "Read through *all* the statements below and then tick the one statement with best describes your current use of tobacco/cigarettes."
    - "*Don't smoke now*, and have smoked less than 100 cigarettes in my whole life."
    - "*Don't smoke now*, but during my life I have smoked more than 100 cigarettes."
    - "Now smoke *occasionally*, but less than once a week."
    - "Now smoke *occasionally*, not everyday, but at least once a week."
    - "Now smoke *regularly*, everyday or most days, about:"
        - "5 or less cigarettes a day"
        - ... (intervals of 5 cigarettes a day)
        - "31 or more cigarettes a day"
2. (1993 if "Don't smoke now", and 1995 all) "Have you ever been a regular smoker?" (Y/N)

In these surveys, the final category in the first question positively identified those who 'currently smoking'. The second question, where available, determined whether an

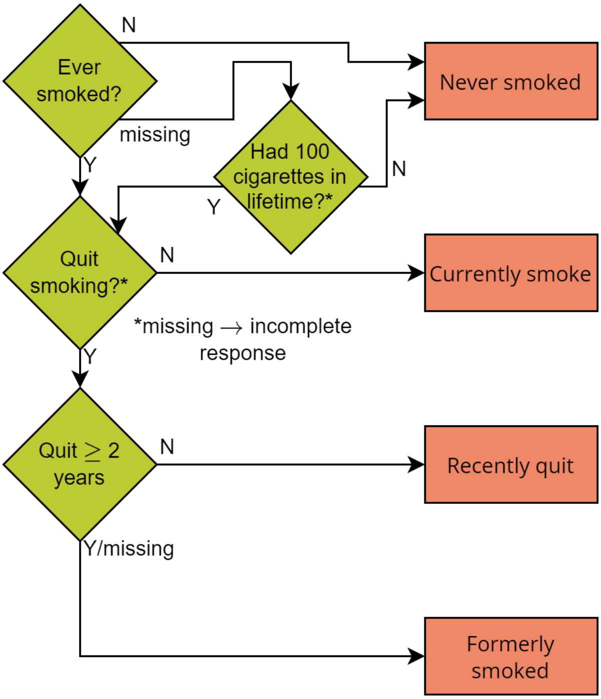

Figure A1. Diagram of participant classification in the National Drug Strategy Household Survey and its predecessors 1985-2019; a similar categorisation applied to the Risk Factor Prevalence Study 1980-89 and the Cancer Council Victoria Adult Smoking Surveys 1974-1995 except a missing response to 'Ever smoked?' was considered an incomplete case.

individual was assigned to 'formerly smoked' (Y) or 'never smoked' (N). Where the second question was not available, those who smoke "occasionally' were assigned to the 'formerly smoked' category. No participants were assigned to 'recently quit' in these surveys due lack of no age-at-quit information.

The RFPS asked:

1. "Have you ever smoked cigarettes, cigars or a pipe regularly?" (Y/N).
2. "Have you given up smoking?"
   - "Yes. I gave up smoking in" [MM/YY]
   - "No I still smoke."

While there is no question on having had 100 cigarettes, all participants could be mapped to one of the categories via mapping these questions to the flow chart.

The CCV Australian adult smoking surveys asked minor variations of the following questions:

1. "Read all the statements. Which one of these statements best describes you?"
   - "Regularly smoke only cigarettes"
   - "Regularly smoke cigarettes and also cigars/pipes"
   - "Regularly smoke cigars, used to smoke cigarettes"
   - "Regularly smoke pipe, used to smoke cigarettes"
   - "Regularly smoke cigars, never smoked cigarettes"
   - "Regularly smoke pipe, never smoked cigarettes"
   - "Don't smoke now, used to smoke only cigarettes"
   - "Don't smoke now, used to smoke cigarettes and pipe/cigars"
   - "Don't smoke now, used to smoke only pipe/cigars"
   - "Never smoked at all"
2. If used to smoke cigarettes about how long ago did you finally give up smoking cigarettes? ($[1, 7]$ days; $[8, 14]$ days; $[15, 21]$ days; $[22, 28]$ days; $[4\text{weeks}, 2\text{months})$; $[2, 3)$ months; $[3, 6)$ months; $[6, 12)$ months; $[1, 2)$ years; $[2, 5)$ years; ≥ 5 years)

No response to the first question was considered an incomplete case. Participants who responded that they regularly smoke any product (the first six options) were assigned to the 'currently smoking' category. Those that "don't smoke now" were assigned 'recently quit' or 'formerly smoked' depending on their time-since-quit category. Otherwise those that responded they never smoked at all were assigned 'never smoked' category. Missing time-since-quit responses for those who had quit smoking were assigned to 'formerly smoked'.

The AGP asked limited questions about tobacco, so participants were classified either as 'currently smoke' or 'do not smoke' (dichotomous).

The survey respondents assigned to 'recently quit' do not necessarily belong to the *model*'s 'recently quit' state which always precedes a persistent (and permanent) cessation of smoking. In other words, some participants who had recently quit will relapse. If all relapse to smoking occurs within two years then the sum of the proportions in the survey's 'currently smoking' and 'recently quit' categories corresponds to the sum of the proportion in the corresponding states of the model; by combining these categories when calculating the survey data likelihood we do not need to explicitly model relapse. In brief, model's quit rate is a reasonable estimate of the permanent quit rate (in the

population) provided that; all individuals initiated smoking before age twenty years; migration was independent of smoking status, and; the model of differential mortality was consistent with the population.

Appendix B Formulation

B.1 Single cohort equations

The categories of smoking status are described in § Model structure (main text). Let: $N(a)$ be the (expected) population size of those who never smoked at age a; $C(a)$ be the size for those who currently smoke; $Q_j(a)$ be the size for those who have recently-quit in age-at-quit category indexed by j, and; $F_j(a)$ and $R_j(a)$ be the sizes for those who formerly smoked (not recently quit) and reporting-as-never smoked respectively.

Using the assumptions outlined in the main text, and indicated in Figure 1 (main text), the mortality rate for those who never smoked and who report as having never smoked was (denoted) μ_N, the rate for those who currently smoke or recently quit was μ_C, and for those who formerly smoked: μ_F. The other transitions in Figure 1 (main text) include: the transition from currently smoke to recently quit, also called the 'quit rate' in the main text, whose rate is denoted λ_Q, and; the transition from formerly smoked to reporting as having never smoked, with rates denoted $\lambda_{R,j}$ for each age-at-quit category indexed by j. The age-at-quit category intervals are given by $[a_j, a_{j+1})$ for each j.

For each smoking status category, the expected population size in a cohort was found by step-wise numerical integration of the delay-differential equations;

$$\frac{\mathrm{d}N}{\mathrm{d}a} = -\mu_N N,$$
$$\frac{\mathrm{d}C}{\mathrm{d}a} = -\left(\lambda_Q + \mu_C\right) C,$$
$$\frac{\mathrm{d}Q_j}{\mathrm{d}a} = \mathbb{I}_{[a_j,a_{j+1})}\lambda_Q C - \left[\mathbb{I}_{[a_j,a_{j+1})}\lambda_Q S_Q C\right]_{a-k} - \mu_C Q,$$

where $S_Q(a) = \exp\left(-\int_a^{a+k} \mu_C(s)\,\mathrm{d}s\right)$ was the survival of individuals that recently quit at age $a+k$ conditional on survival to age a;

$$\frac{\mathrm{d}F_j}{\mathrm{d}a} = \left[\mathbb{I}_{[a_j,a_{j+1})}\lambda_Q S_Q C\right]_{a-k} - \left(\lambda_{R,j} + \mu_F\right) F_j, \text{ and}$$
$$\frac{\mathrm{d}R_j}{\mathrm{d}a} = \lambda_{R,j} F_j - \mu_N R_j,$$

starting with an initial population at age 20 years. The initial population was made up of those who never or currently smoke(d), and those who recently quit and formerly smoked belonging to the first age-at-quit interval (shown in Figure 1 [main text]). The initial number of recent quitters was determined using the following assumptions:

- The recently-quit category was empty $k = 2$ years prior to the initial age, $\hat{a}$;
- No excess mortality occurred in individuals that smoked prior to the initial age;
- The quit rate in the $k = 2$ years prior to the initial age was constant and equal to the quit rate at the initial age.

B.1.1 Integration by quadrature The system of equations, for a given μ_N, was solved by nested Gaussian quadrature rules, using the scale $\exp\left(-\int_{\hat{a}}^{a} \mu_N(s)\,\mathrm{d}s\right)$ to reduce the number of equations by one; the scaled size of the population that never smoked was then trivially a constant. The number who currently smoke, C, was solved by Gauss-Legendre quadrature at each integer age, as the mortality rate was assumed constant within each year. Then, the recently quit and formerly smoked populations, Q_j and F_j, for each age-at-quit category, could be determined at each integer age with Gauss-Legendre quadrature, interpolating the solution of C as needed. Finally, the reporting-as-never population, R_j, was calculated in the same fashion, interpolating F_j as needed.

The value of μ_N was found by simultaneously solving for its relationship to the population mortality μ using the hazard ratios (Equation 1 (main text)). This was achieved via the secant method to a tolerance set close to the size of the error expected in the quadrature rules.

B.2 Spline terms

We denote the initial (at age 20 years) proportion of individuals that formerly smoked, amongst those who ever smoked, as P_F and the proportion of a cohort that initiated as P_I (one minus the proportion of those that never smoked). We modelled the logit-transformed proportion that initiated as a spline-function of the birth year with parameter vector Z_I representing the coefficients of the spline, i.e. for birth year c;

$$\log \frac{P_I(c)}{1 - P_I(c)} = Z_{I,0} + f_I(c; Z_I)$$

for the intercept term $Z_{I,0}$ and f_I a natural cubic spline.

We modelled the log-transformed quit rate as the sum of age and calendar year terms. We denote the coefficients of the respective splines as vectors $Z_{Q,\text{age}}$ and $Z_{Q,\text{year}}$, and

these combined to form the parameter vector $Z_Q = \begin{bmatrix} Z_{Q,\text{age}} & Z_{Q,\text{year}} \end{bmatrix}$. For age a and calendar year p the quit rate λ_Q satisfied;

$$\log \lambda_Q(a, p) = Z_{Q,0} + f_{Q,\text{age}}(a; Z_{Q,\text{age}}) + f_{Q,\text{year}}(p; Z_{Q,\text{year}})$$

for the intercept term $Z_{Q,0}$ and two natural cubic splines $f_{Q,\text{age}}$ and $f_{Q,\text{year}}$.

The log-transformed values of the rate of transition to the reporting-as-never state (λ_R) were denoted as the parameter vector Z_R.

The model parameters were then the (row) vector $Z = \begin{bmatrix} P_F & Z_{I,0} & Z_{Q,0} & Z_I & Z_Q & Z_R \end{bmatrix}$ along with the age-group specific hazard ratios HR_C and HR_F.

The interior knots were placed at equal intervals in the following ranges:

- From year 1910 to 1997 for the birth year-effect spline, f_I, in the proportion that initiate.
- From age 20 to 99 years for the age-effect spline, $f_{Q,\text{age}}$, in the quit rate.
- From years 1930 to 2017 for the calendar year-effect spline $f_{Q,\text{year}}$, in the quit rate.

B.3 Model specifications

We defined eight models with different numbers of knots and with or without the effect that individuals that formerly smoked could report as a never smoking, summarised in Table B1. These models are nested in the sense that the number of parameters describing an effect always increases, but because the knot locations may vary between models they are not nested in the sense that the models lie within subspaces of one another.

B.4 Structural identifiability of the smoking behaviour model

To select identifiable parameters to calibrate we informally examined structural identifiability. We considered the following simplified model for one cohort where;

- individuals that recently quit were indistinguishable from individuals that currently smoked;
- individuals that switched to reporting-as-never were indistinguishable from individuals that never smoked;
- there were no 'age-at-quit' categories, and;
- mortality for individuals that never smoked was zero via an integrating factor.

The first two simplifications reflect the survey data, and we assert that the second two did not materially impact our informal examination. The following equations describe the

Table B1. Specification of each candidate model in the Bayesian calibration of the Australian smoking behaviour model. Shown are the numbers of equi-spaced internal knots in each of the natural cubic splines; the birth year-effect in the proportion that initiate f_I, the age-effect in the quit rate $f_{Q,\text{age}}$, and the calendar year-effect in the quit rate $f_{Q,\text{year}}$, along with whether individuals who quit smoking before age 40 years are able to switch to reporting-as-never.

Model	Description	f_I	$f_{Q,\text{age}}$	$f_{Q,\text{year}}$	Allow report-as-never
**Null**	Constant proportion that initiate, constant quit rate, and no switching to never smoked for those who quit.	0	0	0	No
**A**	As 'Null' +2 d.f. to birth year spline-effect in proportion that initiated.	2	0	0	No
**B**	As 'A' +2 d.f. to age spline-effect in proportion that initiated.	2	2	0	No
**C**	As 'B' +1 d.f. to calendar year spline-effect in quit rate.	2	2	1	No
**D**	As 'C' and allowed those who quit before age 40 years to switch to reporting as had never smoked.	2	2	1	Yes
**E**	As 'D' +1 d.f. to calendar year spline-effect in quit rate.	2	2	2	Yes
**F**	As 'E' +1 d.f. to birth year spline-effect in proportion that initiated.	3	2	2	Yes
**G**	As 'F' +1 d.f. to birth year spline-effect in proportion that initiated.	4	2	2	Yes

age-evolution of the expected proportions in the cohort:

$$\frac{\mathrm{d}N}{\mathrm{d}a} = \lambda_R F,$$
$$\frac{\mathrm{d}C}{\mathrm{d}a} = -\left(\lambda_Q + \mu_C\right) C, \text{ and}$$
$$\frac{\mathrm{d}F}{\mathrm{d}a} = \lambda_Q C - \left(\lambda_R + \mu_F\right) F,$$

where N, C, and F are the expected proportions of never, current and former smoking. We assumed that the ideal outputs are differentiable quantities N, C and F for all values of the continuous age, a. Our informal analysis of identifiability followed: The first equation illustrates that growth in N and the value of F identified the rate of switching. The remaining two equations contained three unknowns λ_Q, μ_C and μ_F, and any values

of these denoted $\tilde{\lambda}_Q$, $\tilde{\mu}_C$ and $\tilde{\mu}_F$, respectively, could be replaced with $\tilde{\lambda}_Q + \epsilon$, $\tilde{\mu}_C - \epsilon$ and $\tilde{\mu}_F - \epsilon C/F$, for any function of age ϵ, and the equations would still be satisfied. Therefore these three parameters were an unidentifiable set. With additional data on at least one of the excess mortality rates, the quit rate (and the other mortality rate) would have been identifiable. The initial state of the cohort was identified by the values of N, C and F at the starting age of the simulation. The identifiable parameters were therefore Z and, at most, one of HR_C or HR_F.

B.5 Detailed prior distribution

We assumed that the joint prior could be decomposed into mutually independent priors for; the hazard ratios (jointly $HR = \begin{bmatrix} HR_C & HR_F \end{bmatrix}$), the proportion P_F, each of the coefficient vectors Z_I and Z_Q, and each rate of switching to reporting-as-never in Z_R.

We used a multivariate normal distribution as an informative prior for the logarithm of the hazard ratios in HR. The mean and covariance of the distribution were provided by Cox regressions using the 45 and Up Study, outlined in the main text, whose untransformed estimates have mean and equal-tailed intervals (ETIs) recorded in Table E3. The analysis was performed with SAS software Version 9.4. The mean was provided by the maximum likelihood estimate (MLE) of the log hazard ratio, and the covariance was estimated by the negative of the inverse of the estimated observed information[9].

We assigned a normal distribution as a prior for $\operatorname{logit}(2P_F)$ with mean $\operatorname{logit}(0.3)$ and $\mathbb{P}(\operatorname{logit} 2P_F < 0.02) = 5\%$. We assigned each rate of switching to reporting as never, λ_R, the prior $1/\sqrt{\lambda_R}$, chosen for being the Jeffreys prior for an exponential random variable, noting that it is *not* a Jeffreys prior given the survey data.

We used multivariate normal priors for the parameter vectors $\begin{bmatrix} Z_{I,0} & Z_I \end{bmatrix}$ and $\begin{bmatrix} Z_{Q,0} & Z_Q \end{bmatrix}$, which provide a small penalty for values away from zero, but were intended to provide little information compared to the survey data. The priors were of the form $\mathcal{N}(0, (X^\top W X)^{-1} w/\sigma^2)$, where X was a basis matrix, W was a diagonal matrix of weights with entries given by the effective sample size for the corresponding row in X, w was the sum of the elements of W, and σ was a nuisance parameter. The value of X for the prior of $\begin{bmatrix} Z_{I,0} & Z_I \end{bmatrix}$ was given by the evaluating the basis functions of the spline f_I at the birth year for individuals that never smoked up to age 25 years, and a column of ones for the intercept term. The value of X for the prior of $\begin{bmatrix} Z_{Q,0} & Z_Q \end{bmatrix}$ was similarly determined by evaluating the basis functions for the sum of the age and calendar effects, $f_{Q,\text{age}}$ and $f_{Q,\text{year}}$, of individuals that smoked in the surveys. Each prior covariance,

$(X^\top WX)^{-1}w/\sigma^2$, was orders of magnitude greater than that of the respective posterior, thus the prior for each spline parameter was suitably uninformative. We assigned each nuisance parameter the prior $1/\sigma$.

B.6 Graphical model of smoking behaviour calibration

We assumed the dependence structure for the calibration as presented in the directed acyclic graph in Figure B2. Parent-child relationships are indicated by directed arrow between nodes in the graph, nodes were conditionally independent of all other nodes except their parents and descendants, and the value of a parents typically 'generated' the values of its children[10]. The nuisance parameters, hazard ratios, and the all-cause mortality data had no parents. The former two were random variables with priors, while the latter was treated as a constant in the model. The model parameters that describe the initial state and the transition rate between states were children of the nuisance parameters. In turn, the initial state and transition rates themselves were children of the parameters, which were governed by the functions discussed in § Model parameterisation (main text). The age-specific population in each state (within a cohort) and the mortality for individuals that never smoked (solved simultaneously as described in § Single cohort equations below) were children of; the rates; the hazard ratios, and; the mortality data. The values of $M(\theta)$, which was the map between the model parameters and the likelihood of the survey data, were given by scaling the expected population to an expected proportion and was a child of the population node. Finally, the survey data was the only child node of the proportions node and was distributed as per § Survey data likelihood.

B.7 Survey data likelihood

An expression for the likelihood function for the survey data would be trivial to formulate were it not for the non-random sampling that occurred either by survey design, e.g. surveys that used multi-stage stratification or quota-sampling designs, or through effects such as non-response. The bias due to non-random sampling has been reduced in reported estimates by weighting the responses. We incorporated the weights for each participant, where supplied, in the following formulation of the likelihood:

- The proportions in each survey-category were vectors, denoted p_i with the index i to each cell in the cross-table of the data by sex, age, birth year and survey.
- Each p_i was independent.

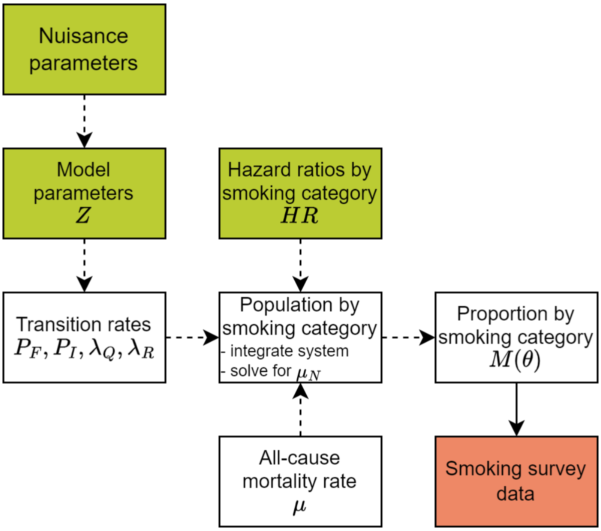

Figure B2. Directed acyclic graph outlining the dependency structure in the calibration of Australian smoking behaviour model. Nodes represent quantities, arrows represent parent-child relationships, and quantities are conditionally independent of all other nodes given their parents and descendants. Model parameters Z, hazard ratios HR, and quantities P_0, P_I, λ_Q, λ_R, are defined in main text. All-cause mortality rate μ treated as constant; μ_N is the never-smoked mortality rate. The values given by $M(\theta)$ correspond to the natural parameters of the likelihood of the smoking survey data.

- Each p_i was observed with the effective sample size $n_{\text{eff},i} = \left(\sum_j w_j\right)^2 / \sum_j (w_j)^2$ where j was the index for each participant (within the ith cell in the cross-table).
- The likelihood for each p_i was:

$$f_{P_i|\Theta}(p_i|\theta) = \text{Dir}\left(M_i(\theta); 1 + p_i n_{\text{eff},i}\right)$$

where $M_i(\theta)$ was the expected value of each proportion for the model by age, sex, and birth year, and Dir denotes the Dirichlet distribution.

We found the expected proportions by numerically solving the governing equations detailed in the preceding section.

The predicted counts of smoking status for a given sex, age and birth year that was sampled n_i times in the survey (where i is the same index as above) were modelled as Dirichlet-Multinomial random variables. We chose the parameters of the distribution for each count such that the variance of the counts would match that of a multinomial with

the effective sample size of the cell, i.e. for the count x_i;

$$f_{X_i|\Theta}(x_i|\theta) = \mathrm{DirMult}(x_i; n = n_i, \alpha = M_i(\theta)\alpha_{0,i}),$$

where $\alpha_{0,i} = n_i(n_{\text{eff},i} - 1)/(n_i - n_{\text{eff},i})$.

Appendix C Numerical methods for practical identifiability

C.1 Numerical evaluation of profile posterior

The profile posterior for the ith component of a parameter θ is the function that maps each value $t \in \mathbb{R}$ to the maximum value of the posterior when $\theta_i = t$, i.e.

$$\begin{aligned}\mathrm{PP}_i(t|x) &= \max_{\theta_{\backslash i}} \mathcal{L}_{X|\Theta_{\backslash i},\Theta_i}\left(x|\theta_{\backslash i}, t\right) f_{\Theta_{\backslash i},\Theta_i}(\theta_{\backslash i}, t),\\ &= \max_{\theta_{\backslash i}} f_{\Theta_{\backslash i},\Theta_i|X}(\theta_{\backslash i}, t|x),\end{aligned}$$

where the subscript $\backslash i$ indicates a vector with its ith component omitted (see[11] equation 2.3).

An estimate $\hat{\theta}_i$ is locally practically identifiable if the posterior-based $1-\alpha$ ‘confidence’ region is finite in extent. This can be determined by considering three cases for the function $\mathrm{PD}_i(t) = 2\,\mathrm{PP}_i(\hat{\theta}_i|x) - 2\,\mathrm{PP}_i(t|x)$, with a threshold Δ_α, which is the $1-\alpha$ quantile of the χ^2 distribution with degrees of freedom equal to the total number of parameters (see[11] Figure 1);

1. Practically (locally) identifiable: A single trough occurs at $\mathrm{PD}_i = 0$ and $t = \hat{\theta}_i$, and the function increases to lie entirely above Δ_a in both directions away from the trough.
2. Practically (locally) non-identifiable: A single trough occurs at $\mathrm{PD}_i = 0$ and $t = \hat{\theta}_i$, and the function increases to lie entirely above Δ_a in at most one direction away from the trough.
3. Structurally (locally) non-identifiable: The function PD_i is flat.

The values of PD_i may be obtained at evenly spaced values, t_j (dropping subscript i), using a predictor-corrector approach. Let $\tilde{\theta}_j$ be the value of θ which maximises the posterior while the ith element is fixed at t_j. Starting from $\tilde{\theta}_0 = \theta_{\text{MAP}}$ and moving away, a prediction $\Delta\tilde{\theta}_{\text{pred}}$ for $\Delta\tilde{\theta}_{j+1} = \tilde{\theta}_{j+1} - \tilde{\theta}_j$ can be found by solving for the local optimum

of the Taylor series approximation to the posterior at $\tilde{\theta}_j$ given by;

$$\mathrm{PP}_i(t_{j+1}|x) \approx \mathrm{PP}_i(t_j|x) + \nabla_\Theta \Delta\tilde{\theta}_{\text{pred}} + \Delta\tilde{\theta}_{\text{pred}}^\top \, \mathrm{H}\left(f_{\Theta|X}(\tilde{\theta}_j|x)\right) \Delta\tilde{\theta}_{\text{pred}},$$

where H is the Hessian matrix operator. The value of H may be approximated via Richardson extrapolation for $j = 0$ (using the `hessian()` function from the numDeriv package in R[12]) and then updated prior to each predictor step for $j \geq 1$ using the symmetric rank-one (SR1) update formula. The local optimum is found by fixing $\Delta\tilde{\theta}_{\text{pred},i} = t_{j+1}$ and equating zero to the gradient of the right-hand side (with respect to $\Delta\tilde{\theta}_{\text{pred},\setminus i}$). The Broyden, Fletcher, Goldfarb and Shanno optimisation algorithm can be used as a corrector step to solve $\Delta\tilde{\theta}_j$ exactly[13]. Using a predictor-corrector approach with SR1 update may reduce computational effort significantly compared to no prediction step ($\Delta\tilde{\theta}_{\text{pred},\setminus i} = 0$).

C.2 Overlap statistic

Another approach to assessing identifiability is to estimate an overlap statistic for each parameter[14,15]. This statistic can be calculated for proper priors, but not improper priors. The overlap statistic for a parameter Θ is:

$$T_{\text{overlap}} = \int_\Theta \min\left(f_\Theta(\theta), f_{\Theta|X}(\theta|x)\right) \,\mathrm{d}\,\theta.$$

When a sufficient sample of the posterior has been obtained, this statistic can be estimated by applying the mid-point algorithm to the kernel density estimate of the posterior. The heuristic value of 0.35 or less can be used as a reference for evidence of 'weak' practical identifiability[14].

Appendix D Metropolis-within-Gibbs sampler

We used the Metropolis-within-Gibbs algorithm to sample from the joint posterior of HR, Z, and the nuisance parameters. Each of these was the basis of a block in the Gibbs sampler. We used the asymptotic distributions of HR at its MLE, and Z and the nuisance parameters at their maximum a posteriori (MAP) estimate, to sample a starting position for each chain, and as the initial covariance for the (normal) jumping distribution for each block. Five chains were simulated, with the starting point sampled from the aforementioned distributions with covariance multiplied by a factor of five. The first

1600 samples were used to determine a scale and covariance for the jumping distribution, targeting an acceptance rate of 0.234, and then discarded. We monitored convergence using the potential variance reduction of the discarded (burn-in) and all samples achieved $\hat{R} < 1.3$. Sampling continued thereon until an estimated effective sample size of 50 was obtained (see[16] §11 equation 11.8). We culled each chain to 40 evenly-spaced samples, for a total of 200 samples from the posterior, to reduce effort in downstream calculations.

The acceptance ratio for each block in the Gibbs sampler was formulated with the aid of the dependence structure shown in Figure B2. The dependence structure simplified the evaluation of the acceptance ratio for the jump in the nuisance parameters as it could be evaluated independently of the likelihood of the survey data.

We outline below the algorithm we used to update the jump distribution for each block at the end of the burn-in phase. We combined the covariance of the burn-in sample with the initial covariance of each block's jump distribution to obtain jump distributions that would sample a normal approximation of the posterior more efficiently.

D.1 Modified burn-in phase for calibration

We modified the Metropolis-within-Gibbs sampler during the burn-in phase to estimate a more efficient jump distribution for each block in the post-burn-in phase. The burn-in phase jump distributions (shared across chains $m = 5$ chains) were fixed within each sub-interval, of width Δ_n, in each chain (we used $\Delta_n = 10$). The block-specific jump distributions in the kth sub-interval were given by zero-centred multivariate normal distributions with covariances equal to estimated block-specific posterior covariances, $\hat{\Sigma}_k$, multiplied by a block-specific factor, $\hat{\beta}_k$. Initial estimates of the posterior covariances were given by $\hat{S}_0 = \Sigma_{\text{MAP}}$, the asymptotic estimate given by the negative inverse Hessian of $f_{\Theta|X}$ evaluated at θ_{MAP}, or, with a similar definition, $\hat{S}_0 = \Sigma_{\text{MLE}}$ for the hazard ratios.

The kth estimates of the block-specific posterior covariances were drawn from the inverse Wishart distribution $W^{-1}(\hat{\Psi}_k, mk\Delta_n)$ where; $\hat{\Psi}_k = \hat{\Psi}_{k-1} + m\Delta_n\hat{S}_{k-1}$; $\hat{\Psi}_0 = 0$; and each $\hat{S}_k$ was given by the pooled sample covariance from the Δ_n samples preceding the $k\Delta_n+$ 1th iteration across all chains. The initial values of β_1 for each block were given by $2.4/\sqrt{d}$ where d was the number of dimensions in the block. Each value of β_k was given by randomly increasing or decreasing the corresponding previous value, based on the probability that the (block-specific) acceptance rate from pooling the Δ_n iterations (preceding the $k\Delta_n+$ 1th iteration) from each chain was greater than 0.234, using Pearson's chi-squared statistic for a proportion.

Once the potential variance reduction measure over the last b sub-intervals (we used $b = 80$) was found to be less than 1.3, a new jump distribution was estimated which would be fixed throughout the post-burn-in phase. The most recent $b\Delta_n$ samples from each chain were pooled to estimate the posterior covariance. The jump distribution's covariance was the estimated posterior covariance multiplied by a point estimate, for an acceptance rate of 0.234, from the regression of β_k onto the obtained acceptance rates in the b preceding sub-intervals.

Appendix E Calibration method and results

The highest confidence level for which the associated posterior-based confidence interval (see Raue et al, 2013[11]) was contained with a given neighbourhood of the *maximum a posteriori* (MAP) estimate is shown Table E2. The neighbourhood was given by $\pm$ 7.1 standard deviations of the asymptotic (normal) distribution of the MAP estimate. The median sampled hazard ratio of the posterior of the all-cause mortality in the Australian population by sex and 5-year age group, by smoking status (reference category: never smoked) is shown in Table E3, along with 90% (ETIs) of the sample, and the median and 90% ETI of the prior, and an estimate of the overlap statistic between the two.

E.1 Deviance information criterion

For each model in Table B1 we estimated the deviance information criterion (DIC). The DIC was estimated using equation 7.9 from Gelman et al, 2013[16] via the expected values of; the hazard ratios, the model parameters, and the log-likelihood. These expectations were taken over the posterior of the model parameters, $\Theta|X$, and this operator was approximated using the posterior samples, denoted HR_j and Z_j, obtained by Markov Chain Monte Carlo. Thus the expected values are denoted $\hat{HR} = \sum_j HR_j/n_{\text{sample}}$, $\hat{Z} = \sum_j Z_j/n_{\text{sample}}$, and $\hat{\mathcal{L}} = \sum_j \mathcal{L}_{X|\Theta}\left(x|\Theta = \begin{bmatrix} HR_j & Z_j \end{bmatrix}\right)/n_{\text{sample}}$, respectively for the hazard ratios, model parameters and log-likelihood. The DIC was then approximated using the following equation:

$$DIC = -4\hat{\mathcal{L}} + 2\mathcal{L}_{X|\Theta}\left(x|\Theta = \begin{bmatrix} \hat{HR} & \hat{Z} \end{bmatrix}\right).$$

The estimate of the DIC for each model is shown in Table E4 by sex. The DIC estimate for model 'F' improved upon all simpler models, and model 'G' only demonstrated a

Table E2. Highest confidence level for which the associated posterior-based confidence interval (see Raue et al, 2013 [11]) of the *maximum a posteriori* (MAP) estimate of a parameter was contained within the neighbourhood given by $\pm$ 7.1 standard deviations of the estimate's asymptotic distribution. Description of each parameter is provided in the main text. Description of each model (labelled Null, and A-G) provided in Table B1. Highest level greater than 95% indicates local practical identifiability.

**Sex**	**Model**	$\log \sigma_{\text{quit}}$	$\log \sigma_{\text{init}}$	$\text{logit } P_{\text{F}}$	$Z_{\text{I},0}$	$Z_{\text{I,cohort},1}$	$Z_{\text{I,cohort},2}$	$Z_{\text{I,cohort},3}$	$Z_{\text{I,cohort},4}$	$Z_{\text{Q},0}$	$Z_{\text{Q,age},1}$	$Z_{\text{Q,age},2}$	$Z_{\text{Q,year},1}$	$Z_{\text{Q,year},2}$	$\log \lambda_{\text{R},1}$	$\log \lambda_{\text{R},2}$
**Women**	Null	84%	95%	>95%	>95%					>95%						
	A	79%	95%	>95%	>95%	>95%	>95%			>95%						
	B	>95%	>95%	>95%	>95%	>95%	>95%			>95%	>95%	>95%				
	C	91%	84%	>95%	>95%	>95%	>95%			>95%	>95%	>95%	>95%			
	D	84%	71%	>95%	>95%	>95%	>95%			>95%	>95%	>95%	>95%		>95%	>95%
	E	84%	65%	>95%	>95%	>95%	>95%			>95%	>95%	>95%	>95%	>95%	>95%	>95%
	F	90%	68%	>95%	>95%	>95%	>95%	>95%		>95%	>95%	>95%	>95%	>95%	>95%	91%
	G	79%	68%	>95%	>95%	>95%	>95%	>95%	>95%	>95%	>95%	>95%	>95%	>95%	>95%	90%
**Men**	Null	84%	94%	>95%	>95%					>95%						
	A	77%	95%	77%	>95%	>95%	>95%			>95%						
	B	>95%	>95%	>95%	>95%	>95%	>95%			>95%	>95%	>95%				
	C	90%	85%	>95%	>95%	>95%	>95%			>95%	>95%	>95%	>95%			
	D	85%	71%	>95%	>95%	>95%	>95%			>95%	>95%	>95%	>95%		>95%	70%
	E	91%	78%	>95%	>95%	>95%	>95%			>95%	>95%	>95%	>95%	>95%	>95%	65%
	F	87%	86%	>95%	>95%	>95%	>95%	>95%		>95%	>95%	>95%	>95%	>95%	>95%	36%
	G	77%	71%	>95%	>95%	>95%	>95%	>95%	>95%	>95%	>95%	>95%	>95%	>95%	>95%	30%

Table E3. Hazard ratio of all-cause mortality in the Australian population by sex and 5-year age groups (45-49 years to 85-89 years) and the age group 90-99 years, by smoking status (referent: never smoked). Shown are the median and the interval given by the 5th and 95th percentiles of; the asymptotic distribution of the maximum likelihood estimate of a Cox proportional hazard model applied to the 45 and Up Study cohort; and the posterior distribution given by the Australian smoking model from the main analysis. Also shown are the overlap statistics between the prior and posterior[14].

		Women			Men		
**Status**	**Age group**	**45 & Up Study prior**	**Posterior**	**Overlap**	**45 & Up Study prior**	**Posterior**	**Overlap**
**Current**	45-49	3.37 [2.53,4.50]	3.15 [2.35,4.20]	0.85	4.01 [3.09,5.21]	3.63 [2.76,4.80]	0.73
	50-54	3.20 [2.54,4.04]	3.11 [2.57,3.87]	0.92	4.07 [3.34,4.95]	3.77 [3.04,4.54]	0.76
	55-59	3.49 [2.83,4.30]	3.72 [2.96,4.51]	0.81	4.02 [3.39,4.78]	3.90 [3.32,4.71]	0.90
	60-64	3.61 [3.00,4.34]	4.01 [3.39,4.93]	0.64	3.64 [3.11,4.26]	3.74 [3.23,4.28]	0.85
	65-69	3.98 [3.32,4.78]	4.75 [3.92,5.59]	0.47	3.14 [2.70,3.65]	3.46 [2.94,4.06]	0.61
	70-74	3.31 [2.69,4.08]	4.20 [3.38,5.02]	0.38	3.18 [2.72,3.72]	3.50 [3.02,4.06]	0.62
	75-79	2.12 [1.67,2.70]	2.56 [2.03,3.28]	0.56	2.42 [2.04,2.88]	2.55 [2.20,2.93]	0.81
	80-84	2.44 [2.00,2.99]	2.45 [1.96,2.90]	0.93	1.96 [1.66,2.32]	1.94 [1.71,2.27]	0.93
	85-89	1.30 [0.97,1.76]	1.14 [0.91,1.52]	0.72	1.55 [1.12,2.13]	1.30 [0.98,1.70]	0.67
	90-99	0.98 [0.50,1.94]	0.96 [0.58,1.64]	0.92	1.54 [0.73,3.24]	0.97 [0.66,1.47]	0.47
**Former**	45-49	1.10 [0.79,1.53]	1.09 [0.79,1.51]	0.95	1.28 [0.94,1.75]	1.26 [0.94,1.78]	0.95
	50-54	1.44 [1.15,1.80]	1.42 [1.09,1.70]	0.93	1.16 [0.93,1.45]	1.15 [0.90,1.37]	0.94
	55-59	1.57 [1.29,1.91]	1.55 [1.24,1.91]	0.93	1.35 [1.14,1.60]	1.30 [1.09,1.55]	0.85
	60-64	1.44 [1.22,1.70]	1.44 [1.22,1.67]	0.95	1.36 [1.19,1.56]	1.34 [1.19,1.53]	0.93
	65-69	1.61 [1.40,1.87]	1.63 [1.39,1.89]	0.94	1.50 [1.33,1.68]	1.51 [1.32,1.72]	0.92
	70-74	1.32 [1.15,1.53]	1.34 [1.17,1.56]	0.92	1.46 [1.31,1.62]	1.48 [1.33,1.64]	0.90
	75-79	1.26 [1.12,1.43]	1.30 [1.16,1.46]	0.86	1.22 [1.11,1.34]	1.25 [1.16,1.37]	0.79
	80-84	1.13 [1.02,1.25]	1.18 [1.07,1.30]	0.78	1.15 [1.07,1.24]	1.18 [1.10,1.26]	0.81
	85-89	1.11 [0.97,1.26]	1.11 [0.99,1.23]	0.94	1.07 [0.96,1.20]	1.17 [0.96,1.17]	0.52
	90-99	1.30 [1.04,1.63]	1.18 [0.99,1.40]	0.70	1.11 [0.92,1.34]	1.12 [0.99,1.28]	0.84

Table E4. Estimated deviance information criterion (see[16] pp. 172-173) for each candidate model in the main analysis.

Model	Description	Women	Men
**Null**	Constant proportion that initiated and quit rate, and no reporting-as-never.	24 666.7	27 813.3
**A**	As ‘Null’ +2 d.f. to birth year spline in proportion that initiated.	24 112.1	24 454.9
**B**	As ‘A’ +2 d.f. to age spline in model of quit rate.	23 476.5	22 613.4
**C**	As ‘B’ +1 d.f. to calendar year spline in model of quit rate.	20 000.3	20 459.6
**D**	As ‘C’ and allowed reporting-as-never for those who quit before age 40 years.	19 217.0	19 681.1
**E**	As ‘D’ +1 d.f. to calendar year spline in quit rate.	19 152.8	19 550.1
**F**	As ‘E’ +1 d.f. to birth year spline in proportion that initiated.	18 714.2	19 504.4
**G**	As ‘F’ +1 d.f. to birth year spline in proportion that initiated.	18 735.9	19 500.2

marginal improvement for men. To minimise risk of over-fitting, we selected model ‘F’ for the main analysis.

E.2 Model discrepancy

We used a Generalised Additive Model to estimate the discrepancy, whose expected value was assumed to be a Gaussian process[17] of the tensor product of age and birth year spline effects. The covariance function of the process was a squared exponential with smoothing parameters estimated by Generalised Cross Validation implemented by the `mgcv` package in R[18,19]. The discrepancy in three values were modelled:

1. The proportion of individuals that smoke in the population from 1962-2016, using all surveys.
2. The proportion of individuals that never smoked amongst those not currently smoking in the population from 1974-2016, excluding the AGP in which individuals not currently smoking could not be differentiated further.
3. The proportion of those who quit before age 30 amongst all those that formerly smoked in the population from 1980-2016 excluding the AGP and CCV surveys, and the NDSHS from 1985-95, which did not supply age-at-quit information.

The mean and standard deviation of the predicted (expected) discrepancy, on a log-odds scale, for the surveyed sample is shown in Table E5, where the expectation was taken over the parameter posterior.

Table E5. Summary of the expected discrepancy in the log-odds of: an individual currently smoking; never smoking if not current; and having quit before age 30 years if formerly smoked, using Generalised Additive Models for the discrepancy fitted to the smoking survey data and the sample of the posterior expected proportions. Summary statistics shown are the estimated mean $\bar{x}$ and standard deviation $\bar{s}$ of the discrepancy.

**Smoking status**		**Current/not current**		**Never/Former**		**Quit $<$ age 30 year**	
**Sex**	**Model**	$\bar{x}$	$\bar{s}$	$\bar{x}$	$\bar{s}$	$\bar{x}$	$\bar{s}$
**Women**	Null	-0.079	0.365	-0.120	0.431	-0.849	0.500
	A	-0.069	0.356	-0.122	0.410	-0.847	0.500
	B	-0.036	0.372	-0.101	0.402	-0.696	0.471
	C	-0.002	0.193	-0.063	0.311	-0.352	0.370
	D	-0.009	0.180	0.004	0.191	-0.126	0.404
	E	-0.012	0.173	-0.001	0.180	-0.129	0.402
	F	-0.008	0.127	-0.008	0.097	-0.114	0.409
	G	-0.008	0.131	-0.008	0.101	-0.118	0.408
**Men**	Null	-0.145	0.497	-0.170	0.328	-1.014	0.428
	A	-0.107	0.279	-0.176	0.380	-1.010	0.427
	B	-0.055	0.285	-0.166	0.356	-0.609	0.403
	C	-0.004	0.161	-0.099	0.317	-0.384	0.312
	D	-0.009	0.143	-0.019	0.185	-0.108	0.343
	E	-0.011	0.120	-0.024	0.171	-0.099	0.332
	F	-0.010	0.116	-0.024	0.153	-0.106	0.330
	G	-0.011	0.114	-0.023	0.152	-0.106	0.331

E.3 Informal validation of quit rate

We defined a quit event as the event that an individual quits and never again smokes (on a daily basis) and in the cross-sectional survey data these events were not distinguishable from ‘attempts’ at quitting with later relapse; therefore we cannot validate the estimate of the model’s quit rate against the proportion of those who smoked within the last year who had maintained abstinence up until the survey. However, in the ITC Four Country Survey, a longitudinal study, it was observed that 95% of those with at least two years abstinence maintained abstinence over the next year[20]. We estimated the proportion of individuals who currently smoke that had quit over a one year period (three years prior to the survey) using the proportion in the survey who had maintained abstinence for at least two years amongst those who either still smoked or had quit within the five years prior to being surveyed, and compared this to the prediction from the model. The survey-estimated proportion is shown in Figure E3 along with the interval given by the 5th and 95th percentiles of the sampled predictions obtained from the selected model. Most of the

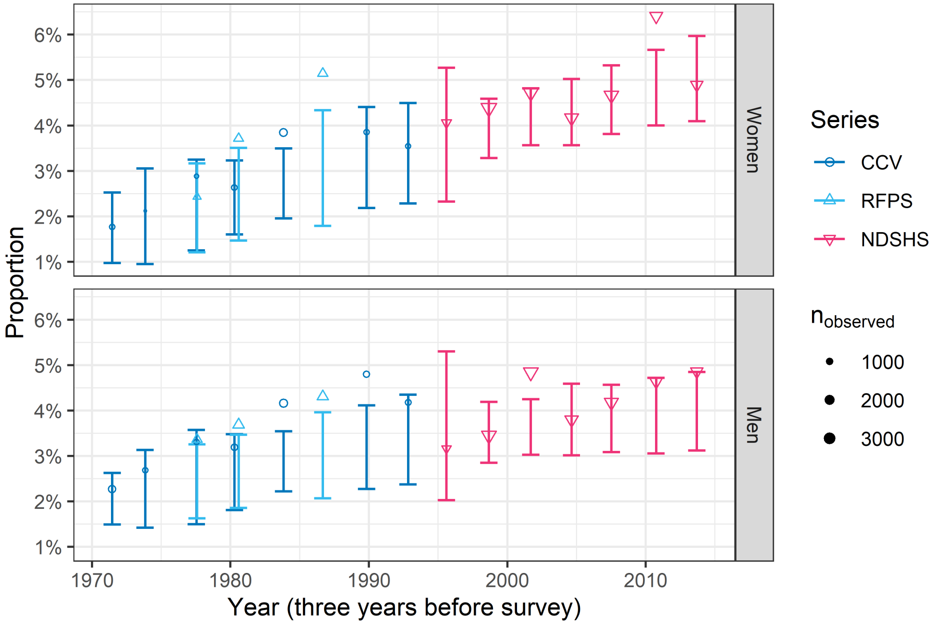

Figure E3. Proportion of Australians who had quit smoking over a one-year period. Shown; the estimate obtained from cross-sectional surveys (markers) using the proportion who were abstinent for at least two years at the time of the survey amongst those who still smoked or had stopped within the five years prior to being surveyed; and the interval given by the 5th and 95th percentiles of the sampled predictions of the proportion obtained from the selected model for each survey (error bars). Size of marker indicates number of respondents who smoked between two and five years prior. Abbreviations; CCV: Cancer Council Victoria adult smoking surveys; RFPS: Risk Factor Prevalence Study; NDSHS: National Drug Strategy Household Survey.

survey-estimated values lie within the intervals, with all outliers being in excess of the upper limit of the interval, which is expected given that the survey estimate would be an over-estimate due to the occurrence of relapse.

Appendix F Sensitivity analyses

In each sensitivity analysis we modified the selected model and obtained a sample from the parameter posterior, estimated the DIC for all but item § Age at which initiation of smoking was completed below as it was not comparable, and we generated expected values of; the sex-specific proportion that initiated by age 20 years in the latest cohort (born 1996), the age and sex-specific rate of quitting daily smoking in the year 2016,

and the sex and age-at-quit group-specific rate of switching from 'former smoking' to reporting-as-never.

F.1 Hazards of smoking-related mortality

Contemporary estimates of the hazards of smoking-related mortality may not be valid throughout the observation period, particularly for earlier years when the hazard ratios are known to be lower due to changes in smoking intensity, age at initiation, and mortality among individuals that never smoke[21]. To assess the sensitivity of the model predictions to the selection of prior for the HRs, we calibrated the selected model using estimates of HRs derived from the 12-year follow-up of the CPS-I cohort[22], recruited in 1959-1960, in the prior instead of the 45 and Up Study estimates. Given that the estimates of the HRs have increased over time in the US[21], this was a prior that greatly under-estimated smoking-related mortality in the later period of the smoking survey data.

F.2 Age at which initiation of smoking was completed

In the survey data, only about 70-85% of individuals who smoked within any birth cohort had started smoking by age 20 years. A more realistic age at completed initiation would be 25 years by which age 93-97% had started[23]. To assess the impact of assuming all smoking starts by age 20 years, we calibrated the selected model using a starting age of 25 years.

F.3 Using a cohort term in the quit rate as opposed to a calendar year term

For simplicity (and identifiability) we chose that the quit rate was a sum of age and calendar year terms, which imply at most a linear relationship with cohort. We considered an alternative in sensitivity analysis by using a sum of age and cohort terms, implying at most a linear calendar year effect. The DIC can be used to determine which choice is more compatible with the survey data, that is, which non-linear effect out of calendar year and cohort is more useful.

F.4 Whether those who quit after age 40 years could switch to reporting-as-never

To assess the sensitivity of the model to the assumption that those who smoked and quit after the age of 40 years would always report that they formerly smoked, and not switch

Table G6. Estimated Deviance Information Criterion of each alternative model in sensitivity analyses, excluding those that are not comparable to the main analysis (the alternative of no survey weights, and the alternative initial age of 25 years).

**Description**	**Women**	**Men**
Selected model	18 714.2	19 504.4
Use HR of smoking-related death observed in Cancer Prevention Study I rather than 45 and Up Study	18 848.1	19 565.7
Allow those who quit after age 40 years to switch to reporting-as-never	18 702.0	19 505.3
No reporting-as-never permitted	19 358.3	20 130.0
Use cohort instead of calendar year in quit rate	18 781.8	19 607.0

to reporting as never, we calibrated the selected model with the assumption of a non-zero rate of switching for the later age-at-quit group. Similarly, we tested no reporting as a never for all age-at-quit groups if the selected model did allow switching.

F.5 Ignoring survey weighting

In the main analysis we used the approximate effective sample size of the smoking survey data and individual weights to account for survey design effects. To determine how sensitive our results were to the adjustment for non-random sampling, we also calibrated the selected model ignoring the weights and effective sample size in the likelihood.

Appendix G Sensitivity analyses results

Table G7. Sample obtained from the selected model and the sensitivity analyses of the proportion in an Australian birth cohort that initiated daily smoking for birth year 1996 by sex. Shown are the sample median and the interval given by the 5th and 95th percentiles of each sample.

Description	Women	Men
Selected model	16.2% [15.3%,17.2%]	22.4% [21.0%,24.0%]
Use HR of smoking-related death observed in Cancer Prevention Study I rather than 45 and Up Study	16.3% [15.2%,17.5%]	22.5% [21.2%,23.8%]
Use cohort instead of calendar year in quit rate	16.4% [15.3%,17.4%]	22.4% [21.2%,23.8%]
Allow those who quit after age 40 years to switch to reporting-as-never	16.4% [15.5%,17.7%]	22.4% [20.9%,23.9%]
No reporting-as-never permitted	17.4% [16.4%,18.5%]	22.9% [21.5%,24.3%]
Ignore weights for smoking survey responses in likelihood	18.7% [17.7%,19.8%]	23.8% [22.7%,25.3%]

Table G8. Sample obtained from the selected model and a sensitivity analysis, where the age at completed initiation was raised to 25 years, of the proportion in an Australian birth cohort that initiated daily smoking for birth year 1991 by sex. Shown are the sample median and the interval given by the 5th and 95th percentiles of each sample.

Description	Women	Men
Selected model	22.7% [21.8%,23.6%]	27.7% [26.5%,29.0%]
Raise age at complete initiation to 25 years	22.3% [21.1%,23.7%]	31.1% [29.4%,33.1%]

Table G9. Samples obtained from the selected model and the sensitivity analyses of the rate that Australians quit daily smoking (per 100 person-years) in the calendar year 2016 by age at 30, 50 and 70 years, and by sex. Shown are the sample median and the interval given by the 5th and 95th percentiles of each sample.

	Age 30 years		Age 50 years		Age 70 years	
**Description**	**Women**	**Men**	**Women**	**Men**	**Women**	**Men**
Selected model	5.52 [5.18,5.86]	3.66 [3.44,3.90]	5.08 [4.80,5.30]	4.71 [4.46,5.02]	5.89 [5.22,6.48]	4.78 [4.16,5.35]
Use HR of smoking-related death observed in Cancer Prevention Study I rather than 45 and Up Study	5.50 [5.21,5.82]	3.62 [3.41,3.84]	5.20 [4.95,5.49]	4.81 [4.60,5.07]	7.80 [7.13,8.49]	6.48 [6.00,7.01]
Raise age at complete initiation to 25 years	6.61 [6.22,7.04]	4.16 [3.86,4.46]	4.95 [4.67,5.25]	4.67 [4.40,4.97]	6.87 [6.23,7.57]	5.47 [4.90,6.06]
Use cohort instead of calendar year in quit rate.	6.71 [6.34,7.00]	4.70 [4.42,5.02]	6.06 [5.80,6.30]	5.95 [5.72,6.21]	7.59 [6.80,8.32]	6.86 [6.23,7.49]
Allow those who quit after age 40 years to switch to reporting-as-never	5.46 [5.11,5.75]	3.65 [3.42,3.87]	5.26 [5.00,5.56]	4.69 [4.46,4.99]	6.81 [6.04,7.44]	4.88 [4.38,5.52]
No reporting-as-never permitted	5.21 [4.88,5.50]	3.36 [3.16,3.56]	5.26 [5.00,5.56]	4.88 [4.62,5.17]	6.66 [5.98,7.26]	4.60 [4.23,5.13]
Ignore weights for smoking survey responses in likelihood	5.52 [5.24,5.87]	3.45 [3.26,3.63]	5.47 [5.16,5.76]	4.51 [4.33,4.72]	6.45 [5.88,7.07]	4.82 [4.40,5.32]

Table G10. Samples obtained from the selected model and the sensitivity analyses of the rate that individuals that formerly smoked switch to reporting-as-never (per 100 person-years) by age-at-quit group and sex. Shown are the sample median and the interval given by the 5th and 95th percentiles of each sample.

Description	Age quit 0-29 years		Age quit 30-39 years		Age quit 40-99 years	
	**Women**	**Men**	**Women**	**Men**	**Women**	**Men**
Selected model	2.33 [2.17,2.51]	2.05 [1.92,2.18]	0.85 [0.68,1.05]	0.29 [0.14,0.45]	-	-
Use HR of smoking-related death observed in Cancer Prevention Study I rather than 45 and Up Study	2.34 [2.20,2.49]	2.06 [1.93,2.18]	0.79 [0.63,0.98]	0.20 [0.09,0.36]	-	-
Raise age at complete initiation to 25 years	2.72 [2.58,2.85]	2.33 [2.21,2.50]	0.96 [0.73,1.15]	0.36 [0.18,0.51]	-	-
Use cohort instead of calendar year in quit rate	2.47 [2.30,2.61]	2.22 [2.12,2.37]	0.91 [0.74,1.13]	0.29 [0.18,0.44]	-	-
Allow those who quit after age 40 years to switch to reporting-as-never	2.28 [2.15,2.43]	2.05 [1.91,2.18]	0.87 [0.68,1.09]	0.28 [0.14,0.43]	0.67 [0.40,0.92]	0.04 [0.00,0.17]
Ignore weights for smoking survey responses in likelihood	2.42 [2.30,2.54]	2.05 [1.94,2.18]	0.65 [0.48,0.82]	0.16 [0.04,0.26]	-	-

Table G11. Age-standardised sex-specific hazard ratio of death from all causes by smoking status (compared to never smoked) in each sensitivity analyses and the prior ratio given by the proportional hazards model of the 45 and Up Study. Hazard ratios were standardised to the sex-specific age-distribution in the Australian population from calendar year 2000 (Human Mortality Database[24]). Shown are; the sample median and the interval given by the 5th and 95th percentiles of each posterior sample in the sensitivity analyses and the selected model of the main analysis; the same percentiles for the prior distribution; and the estimated overlap statistics compared to the prior distribution[14].

	Smoking Status	Current		Former	
Sex	Description	Hazard ratio	Overlap	Hazard ratio	Overlap
Women	Prior (45 and Up Study)	3.21 [2.93,3.52]	-	1.36 [1.26,1.48]	-
	Selected model	3.42 [3.16,3.72]	0.57	1.35 [1.24,1.49]	0.92
	Raise age at complete initiation to 25 years	3.37 [3.13,3.68]	0.61	1.35 [1.25,1.45]	0.95
	Use cohort instead of calendar year in quit rate	3.37 [3.10,3.72]	0.65	1.36 [1.27,1.48]	0.96
	Allow those who quit after age 40 years to switch to reporting-as-never	3.31 [3.01,3.59]	0.80	1.34 [1.25,1.46]	0.89
	No reporting-as-never permitted	3.53 [3.26,3.83]	0.39	1.48 [1.34,1.61]	0.40
	Ignore weights for smoking survey responses in likelihood	3.33 [3.10,3.59]	0.72	1.35 [1.23,1.44]	0.93
Men	Prior (45 and Up Study)	3.60 [3.30,3.92]	-	1.31 [1.21,1.44]	-
	Selected model	3.51 [3.27,3.79]	0.81	1.31 [1.20,1.43]	0.93
	Raise age at complete initiation to 25 years	3.43 [3.15,3.70]	0.66	1.28 [1.18,1.40]	0.81
	Use cohort instead of calendar year in quit rate	3.44 [3.19,3.72]	0.68	1.32 [1.20,1.45]	0.93
	Allow those who quit after age 40 years to switch to reporting-as-never	3.45 [3.22,3.73]	0.93	1.29 [1.20,1.41]	0.86
	No reporting-as-never permitted	3.60 [3.39,3.95]	0.93	1.44 [1.33,1.56]	0.38
	Ignore weights for smoking survey responses in likelihood	3.30 [3.06,3.53]	0.37	1.28 [1.18,1.39]	0.82

References

1. Australian Institute of Health and Welfare. National Drug Strategy Household Survey 2016: detailed findings. Technical report, AIHW, Canberra, 2017. DOI:10.25816/5ec5bc1bed176.
2. McAllister I. The 1985-1993 NDS surveys: measuring patterns of alcohol and tobacco use. In Dillon P (ed.) *The National Drug Strategy: the first ten years and beyond: proceedings from the eighth National Drug and Alcohol Research Centre Annual Symposium*. University of New South Wales: National Drug and Alcohol Research Centre, pp. 17–33.
3. Risk Factor Prevalence Study Management Committee. *Risk factor prevalence study. Survey No 3 1989*. Canberra: National Heart Foundation of Australia and Australian Institute of Health, 1990.
4. Hill DJ, White VM and Scollo MM. Smoking behaviours of Australian adults in 1995: trends and concerns. *Medical Journal of Australia* 1998; 168(5): 209–213. DOI:10.5694/j.1326-5377.1998.tb140132.x.
5. Australian Gallup Opinion Polls. Australian gallup polls subscriber reports. Technical report, Australian Public Opinion Polls, Melbourne, 1962.
6. Australian Gallup Opinion Polls. Australian gallup polls subscriber reports. Technical report, Australian Public Opinion Polls, Melbourne, 1963.
7. Australian Gallup Opinion Polls. Australian gallup polls subscriber reports. Technical report, Australian Public Opinion Polls, Melbourne, 1964.
8. Australian Gallup Opinion Polls. Australian gallup polls subscriber reports. Technical report, Australian Public Opinion Polls, Melbourne, 1968.
9. SAS Institute Inc. *SAS/STAT ©15.1 User's Guide*. SAS Institute Inc, Cary, NC, 2018.
10. Jackson CH, Jit M, Sharples LD et al. Calibration of complex models through Bayesian evidence synthesis: a demonstration and tutorial. *Medical Decision Making* 2015; 35(2): 148–161. DOI:10.1177/0272989X13493143.
11. Raue A, Kreutz C, Theis FJ et al. Joining forces of Bayesian and frequentist methodology: a study for inference in the presence of non-identifiability. *Philosophical Transactions of the Royal Society A: Mathematical, Physical and Engineering Sciences* 2013; 371(1984): 20110544. DOI:10.1098/rsta.2011.0544.
12. Gilbert P and Varadhan R. numDeriv: Accurate numerical derivatives, 2019. URL https://CRAN.R-project.org/package=numDeriv. R package version 2016.8-1.1.
13. Nocedal J and Wright S. *Numerical optimization*. Second edition ed. New York: Springer, 2006. DOI:10.1007/978-0-387-40065-5.
14. Garrett ES and Zeger SL. Latent class model diagnosis. *Biometrics* 2000; 56(4): 1055–1067. DOI:10.1111/j.0006-341X.2000.01055.x.

15. Rutter CM, Miglioretti DL and Savarino JE. Bayesian calibration of microsimulation models. *Journal of the American Statistical Association* 2009; 104(488): 1338–1350. DOI: 10.1198/jasa.2009.ap07466.
16. Gelman A, Carlin JB, Stern HS et al. *Bayesian data analysis*. Third ed. New York: Chapman and Hall/CRC, 2013. DOI:10.1201/b16018.
17. Kammann EE and Wand MP. Geoadditive models. *Journal of the Royal Statistical Society: Series C (Applied Statistics)* 2003; 52(1): 1–18. DOI:10.1111/1467-9876.00385.
18. Wood SN. Low-rank scale-invariant tensor product smooths for Generalized Additive Mixed Models. *Biometrics* 2006; 62(4): 1025–1036. DOI:10.1111/j.1541-0420.2006.00574.x.
19. Wood SN. *Generalized Additive Models: an introduction with R*. Second ed. Boca Raton: Chapman and Hall/CRC, 2017. DOI:10.1201/9781315370279.
20. Herd N, Borland R and Hyland A. Predictors of smoking relapse by duration of abstinence: findings from the International Tobacco Control (ITC) Four Country Survey. *Addiction* 2009; 104(12): 2088–2099. DOI:10.1111/j.1360-0443.2009.02732.x.
21. Thun MJ, Carter BD, Feskanich D et al. 50-year trends in smoking-related mortality in the United States. *New England Journal of Medicine* 2013; 368(4): 351–364. DOI: 10.1056/NEJMsa1211127.
22. Burns DM, Shanks TG, Choi W et al. The American Cancer Society Cancer Prevention Study I: 12-year followup of 1 million men and women. In Burns DM, Garfinkel L and Samet JM (eds.) *Changes in cigarette-related disease risks and their implications for prevention and control*, *Tobacco Control Monograph*, volume 8. Bethesda: National Cancer Institute, 1997. pp. 113–304.
23. Vaneckova P, Wade S, Weber M et al. Birth-cohort estimates of smoking initiation and prevalence in 20th century Australia: Synthesis of data from 33 surveys and 385,810 participants. *PLOS ONE* 2021; 16(5): 1–17. DOI:10.1371/journal.pone.0250824.
24. HMD. Human mortality database, 2020. URL www.mortality.org.
